# Distinguishing between Incomplete Lineage Sorting and Genomic Introgressions: Complete Fixation of Allospecific Mitochondrial DNA in a Sexually Reproducing Fish (*Cobitis*; Teleostei), despite Clonal Reproduction of Hybrids

**DOI:** 10.1371/journal.pone.0080641

**Published:** 2014-06-27

**Authors:** Lukas Choleva, Zuzana Musilova, Alena Kohoutova-Sediva, Jan Paces, Petr Rab, Karel Janko

**Affiliations:** 1 Laboratory of Fish Genetics, Institute of Animal Physiology and Genetics, AS CR, v.v.i., Libechov, Czech Republic; 2 Ecological Genetics Research Unit, Department of Biosciences, University of Helsinki, Helsinki, Finland; 3 Zoological Institute, Evolutionary Biology, University of Basel, Basel, Switzerland; 4 Department of Zoology, Faculty of Science, Charles University in Prague, Prague, Czech Republic; 5 Institute of Zoology, Slovak Academy of Sciences, Bratislava, Slovakia; 6 Laboratory of Genomics and Bioinformatics, Institute of Molecular Genetics of the ASCR, v.v.i., Prague, Czech Republic; 7 Life Science Research Centre, Department of Biology and Ecology, Faculty of Natural Sciences, University of Ostrava, Ostrava, Czech Republic; University of Massachusetts, United States of America

## Abstract

Distinguishing between hybrid introgression and incomplete lineage sorting causing incongruence among gene trees in that they exhibit topological differences requires application of statistical approaches that are based on biologically relevant models. Such study is especially challenging in hybrid systems, where usual vectors mediating interspecific gene transfers - hybrids with Mendelian heredity - are absent or unknown. Here we study a complex of hybridizing species, which are known to produce clonal hybrids, to discover how one of the species, *Cobitis tanaitica*, has achieved a pattern of mito-nuclear mosaic genome over the whole geographic range. We appplied three distinct methods, including the method using solely the information on gene tree topologies, and found that the contrasting mito-nuclear signal might not have resulted from the retention of ancestral polymorphism. Instead, we found two signs of hybridization events related to *C. tanaitica*; one concerning nuclear gene flow and the other suggested mitochondrial capture. Interestingly, clonal inheritance (gynogenesis) of contemporary hybrids prevents genomic introgressions and non-clonal hybrids are either absent or too rare to be detected among European *Cobitis*. Our analyses therefore suggest that introgressive hybridizations are rather old episodes, mediated by previously existing hybrids whose inheritance was not entirely clonal. *Cobitis* complex thus supports the view that the type of resulting hybrids depends on a level of genomic divergence between sexual species.

## Introduction

Interspecific genetic exchange has long been recognized as an important feature in the evolution of plants, but a growing amount of recent studies suggest that hybridization may be potentially important in animal evolution as well, as it can occasionally lead to the formation of new species [1]–[5]. Hybridization is often inferred from topological incongruence between gene trees. A major problem in correctly detecting hybridization lies in the fact that conflicting phylogenetic signals among loci may also be caused by other processes, namely incomplete lineage sorting. Traditional phylogenetic approaches based on independent inspection of several loci and comparison of the geographic distribution of different lineages may help to identify potential cases of discordant evolution [6]–[8]; however, they cannot rigorously test the two processes that result in discordant genealogies.

Although distinguishing hybrid introgression from incomplete lineage sorting remains a critical task in evolutionary studies, yet no effective and widely applicable approach exists for distinguishing these processes [9]. However, recent methodological advances in the development of so called ‘coalescent genealogy samplers’ [3], [10] have greatly facilitated the use of multiple loci as the basis for estimating the sizes of diverging populations, time since their divergence, and immigration rates in a statistical framework [3], [11]–[14]. Nevertheless, each statistical method is based on some simplifying assumptions (e.g. constant population size, marker neutrality, constant immigration rate through time) that may in fact be violated, making the biologically relevant interpretation of obtained results challenging. Therefore, knowledge of the studied organism is essential in understanding how differences between the real population and its simplified representation can affect the results of the analysis [3], [10], e.g. in order to make a proper assessment of the origin, extent and evolutionary significance of hybrid introgression.

Introgressive hybridization arises if two species that come into contact – either throughout the process of speciation [2], [15], [16] or by secondary contact [8] – are not completely reproductively isolated. Here, the asymmetry in levels of genetic introgression between hybridizing species appears to be the rule rather than exception. The phenomenon is caused by the fact that the permeability of interspecific barriers differs among genomic regions. In particular, mtDNA is known to easily introgress into allospecific gene pools. This behavior may be explained by an interplay of selection [17]–[19] and hereditary characteristics; specifically, the maternal inheritance and smaller effective population size of mtDNA suggest that a recipient population may receive foreign mtDNA more easily than nuclear genes [20]–[23]. On some occasions, ancestral mtDNA haplotypes may even be completely replaced by the introgressed ones in the apparent absence of nuclear introgression [24]–[26]. On the other hand, contrasting cases have documented the presence of nuclear gene flow with no mitochondrial introgressions [27]. In addition, many hybrid species carry mosaic genotypes in nuclear DNA while their mtDNA is derived from one or both parental taxa [1], [4], [28], [29]. This ultimately leads to the realization that a single gene tree genealogy may or may not reflect the true history of a species (tree). Hence, combining multiple independent nuclear DNA and mtDNA markers, along with incorporating intraspecific sampling of several individuals [30] coupled with good geographical coverage of the species of interest, improves the power to detect the genetic pattern in current species and to test for hybridization in the presence of coalescence.

Model-based inferences of historical gene flow among sexually reproducing species are greatly enhanced by direct evidence for hybridizations. Such hybrids have usually achieved the independent segregation of parental chromosomes during meiosis. Their ongoing hybridizations result in various types of hybrid introgressions in populations [31]. Therefore, fertile, sexually reproducing hybrids are usually assumed as key players in genomic introgressions (e.g. [5], [32]), because they backcross to sexual species and thus mediate a “bridge” for DNA introgression from one species to another. However, instances of conflicting gene genealogies have also been detected in animal systems where sexual species produce hybrids that lack regular meiotic cycles and whose reproduction deviates from the canonical rules of heredity [33]–[40]. Clonal reproduction, either in the absence of fertilization (parthenogenesis) or syngamy (gynogenesis; e.g. the sperm is only used to trigger the egg development), maintains hybrids in a permanent F_1_ state, thereby preventing gene introgression into sexual species. When genetic interaction with sexual species occurs, it is known to involve either true fertilization that increases the ploidy level of the hybrid lineage [41], or incorporations of parts of the sperm genome persisting as microchromosomes [42]. Nonetheless, some animals combine clonally and sexually transmitted genomes using so-called ‘quasi-sexual reproductive modes’ – e.g. hybridogenesis [41], meiotic hybridogenesis [37], [37], [43]–[45], kleptogenesis [46], or pre-equalizing hybrid meiosis [47]. For instance, hybridogenetic hybrids can mediate rapid introgression of mtDNA or nuclear genome *en bloc* from one species to another without meiotic admixture of parental genomes (nuclear hybridity), producing a clear mito-nuclear mosaic genome [37]. Hence, investigations of animals displaying both sexual and non-sexual heredity could provide important insights into unknown processes of reticulate evolution, and identify potential drivers of reticulate events [48]. Here we focus on a freshwater fish of the genus *Cobitis*, and document the existence of one of the most geographically widespread examples of mito-nuclear mosaic genome among animals, and further investigate its origin.

**Figure 1:**
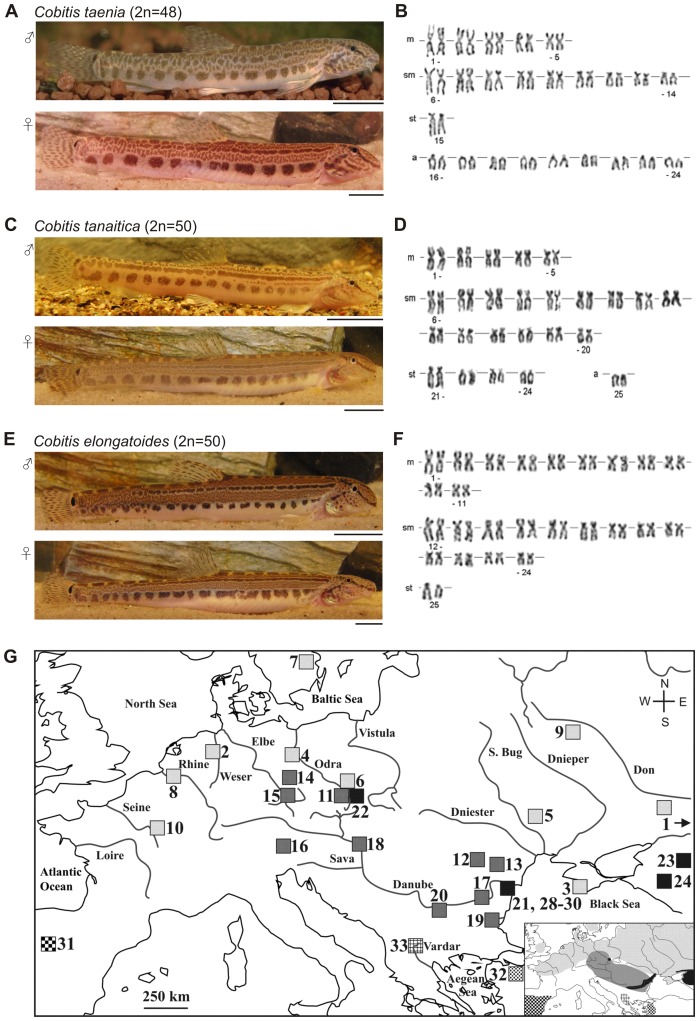
Photographs, karyotypes and Europe-wide distribution of spined loach sexual species (*Cobitis*) from this study. (A,C,E) Photographs (scale bar = 1 cm) and (B,D,F) respective karyotypes of three widespread *Cobitis* species. Karyograms with diploid chromosome number (2n), metacentric (m), submetacentric (sm), subtelocentric (st), and acrocentric chromosomes (a) were modified after Janko et al. [50]; (G) Sampling localities of *Cobitis taenia* (light gray squares; 1–10), *C. elongatoides* (dark gray squares; 11–20), *C. tanaitica* (black squares; 21–30), *C. paludica* (checkered square; 31), *C. fahirae* (spotted square; 32), and *C. vardarensis* (reticulated square; 33). Insets show European species distribution with respective markings as given in squares. Note that locality no. 1 is situated more eastward, as marked by the arrow.

The so-called ‘*Cobitis taenia* hybrid complex’ comprises several sexual species having a parapatric distribution, of which three species show closely adjacent ranges in non-Mediterranean Europe: *C. elongatoides*, *C. tanaitica*, and *C. taenia* (Figure 1A–G). *C. elongatoides* (diploid chromosome number 2n = 50) and *C. taenia* (2n = 48) are well defined by karyotype (Figure 1D,F) and mito-nuclear markers [49]–[51]. The third sexual species, *C. tanaitica*, (2n = 50) is well distinguishable from the other two species by karyotype (Figure 1B,D,F; [50]).

We showed previously [52] that primary hybridizations take place between the species *C. elongatoides* and *C. taenia* in narrow hybrid zones in the upper Elbe, Odra Rivers and in the northern Black Sea shelf. Population genetic analyses of the Odra R. hybrid zone revealed fixed heterozygotes as the only form of hybrids [53]. Laboratory crossing experiments confirmed strictly clonal gynogenetic reproduction of hybrid females, while hybrid males were infertile [54], [55]. Paternal leakage of subgenomic amounts has never been observed, but incorporations of entire chromosome sets into diploid or triploid eggs result in the formation of a new polyploid gynogenetic lineage [54], [55]. Clonal hybrids are now distributed throughout most of Europe, including most of the distribution ranges of the parental taxa – even in allopatric areas. Hybrids between *C. elongatoides* and *C. tanaitica* are also known to occur as fixed clonal heterozygotes appearing as di, tri- and tetraploids. They have arisen in the lower Danube and spread over most of the Balkan Peninsula and Central Europe [50], [56], [57]. Hence, speciation between sexual species of spined loaches seems virtually complete despite their reproductive contact during the Pleistocene/Holocene era, because their diploid and polyploid hybrids are permanent F_1_ heterozygotes and reproduce clonally via gynogenesis [49], [50], [54], [55], [57]–[60]. Clonal heredity of hybrids should therefore prevent any introgressive hybridization. However, recent findings suggest that one of the parental species, *C. tanaitica*, is a genetic mosaic because its mtDNA clusters exclusively with *C. elongatoides* in the whole distribution range including distant allopatric regions [49], see also Figure 1G) but its nuclear allozymic markers are almost indistinguishable from those of *C. taenia* [48].

In the present study, we sequenced multiple single-copy nuclear loci and the mitochondrial cytochrome *b* locus to test if nuclear and mtDNA loci are in topological conflict within the three-taxa model. We subsequently tested whether the observed patterns may be explained by incomplete lineage sorting or by massive hybrid introgression. The latter case would indicate unusual permeability of species boundaries despite the fact that most contemporary hybridization events lead to clonally reproducing hybrids. Because the detection of interspecific gene flow, which is based solely on statistical models, is rather delicate in cases when traditional hybrids mediating gene introgression through Mendelian heredity are unknown, we simultaneously applied several analytical approaches in order to be confident that the observed signal of interspecific gene flow is not an artifact of any particular method. Finally, we propose hybrid speciation scenarios explaining the origin of *C. tanaitica*, related to general understanding the extent to which gene flow persists throughout the process of animal speciation through time.

## Results

### Sequence Variability, Neutrality Tests, Structure, and Divergence

The levels of nuclear DNA and mtDNA sequence polymorphism in *Cobitis* species are summarized in Table 1. Overall, the nucleotide variation in nuclear gene markers was much lower compared to the mitochondrial *cytb* locus. Tajima's relative rate test for all species pairs versus outgroup comparisons did not reject the null hypothesis of equal rates in all loci (*p*>0.15]. The *RpS7* and *RAG1* genes were inferred to have up to three recombination events and hence only the largest non-recombinant blocks of both loci were selected for the coalescence-based analyses.

**Table 1:** Summary of nucleotide variation.

Locus^a^	*L*	*h*	*S*	*Indels*	*Haplotype diversity ± SD*	*Nucleotide diversity ± SD*
***28S***	280	1, 1, 1, 2	0, 0, 0, 1	0	0.000±0.000, 0.000±0.000, 0.000±0.000, 0.452±0.042	0.000±0.000, 0.000±0.000, 0.000±0.000, 0.002±0.001
***Act-2***	306–330	3, 3, 2, 7	2, 3, 2, 7	0, 2, 0, 2	0.626±0.104, 0.530±0.136, 0.556±0.075, 0.802±0.042	0.003±0.001, 0.002±0.002, 0.003±0.002, 0.005±0.002
***Atp-B***	206–212	1, 5, 2, 7	0, 4, 2, 8	1, 0, 1, 1	0.000±0.000, 0.632±0.088, 0.189±0.108, 0.563±0.065	0.000±0.000, 0.006±0.003, 0.002±0.002, 0.009±0.003
***N2***	435–514	1, 5,1, 6	0, 7,0, 10	0, 2, 0, 2	0.000±0.000, 0.758±0.077, 0.000±0.000, 0.533±0.071	0.000±0.000, 0.003±0.001, 0.000±0.000, 0.008±0.001
***N4***	278–648	1, 3, 1, 5	0, 2, 0, 15	1, 1, 1, 2	0.000±0.000, 1.000±0.272, 0.000±0.000, 1.000±0.126	0.000±0.000, 0.002±0.001, 0.000±0.000, 0.014±0.003
***N6***	548–567	3, 2, 4, 7	2, 2, 3, 13	1, 0, 1, 1	0.582±0.142, 0.200±0.154, 0.773±0.083, 0.801±0.037	0.002±0:001, 0.001±0.001, 0.002±0.001, 0.008±0.001
***Rag1***	653	5, 6, 2, 13	3, 7, 7,11	0	0.711±0.085, 0.837±0.047, 0.837±0.047, 0.820±0.032	0.001±0.001, 0.005±0.001, 0.005±0.001, 0.005±0.001
***Rhod***	507–515	1, 2, 1, 3	0, 1, 0, 7	0	0.000±0.000, 0.100±0.088, 0.000±0.000, 0.463±0.047	0.000±0.000, 0.000±0.000, 0.000±0.000, 0.005±0.001
***RpS7***	575–592	7, 3, 3, 13	4, 3, 4, 29	1, 4, 3, 6	1.000±0.076, 1.000±0.272, 1.000±0.177, 1.000±0.027	0.003±0.001, 0.003±0.002, 0.004±0.002, 0.019±0.003
***cytb***	1088	5, 10, 8, 23	9, 19, 20, 78	0	1.000±0.126, 1.000±0.045, 1.000±0.063, 1.000±0.013	0.003±0.001, 0.005±0.001, 0.007±0.002, 0.021±0:002

a Data are in the order for *C. taenia*; *C. elongatoides*; *C. tanaitica*; and all three species. *Cobitis fahirae*, *C. vardarensis* and *C. paludica* were sequenced as one individual per species per locus and not summarised. *L*, sequence length (bp); *h*, number of haplotypes; *S*, number of polymorphic sites.

The Hudson-Kreitman-Aguade test for the nuclear dataset alone was insignificant, but inclusion of the *cytb* sequences resulted in a significant outcome (*p* = 0.006). The *cytb* locus exhibited high intraspecific and low interspecific variability. Tachida's *Z* was not significant for any coding nuclear loci, but was significantly negative for *cytb*, suggesting the presence of purifying selection. As noted, e.g., by Nadachowska and Babik [61], directional and balancing selection is likely to invalidate the analyses of interspecific gene flow, whereas purifying selection only decreases the overall mutation rate. Therefore, we assume that our data are suitable for subsequent analyses, because we observed no evidence of balancing or directional selection.

**Figure 2:**
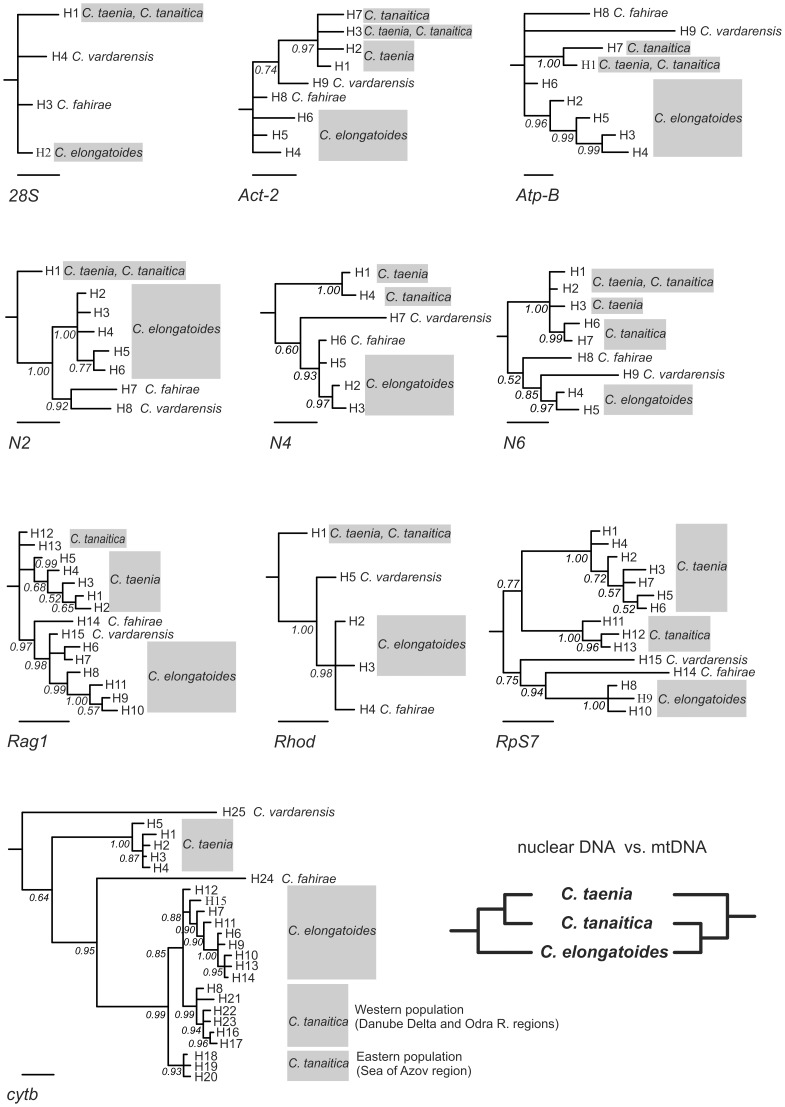
Phylogenetic comparison of gene trees constructed from nuclear and mitochondrial gene markers and mito-nuclear discordance. Bayesian DNA gene trees constructed from nine nuclear gene markers and one mitochondrial *cytb* gene marker were rooted with sequences from *C. paludica*. Haplotype numbers correspond to Table 2. Bar represents 0.1 substitution/site. The schematic tree shows the phylogenetic conflict of *C. tanaitica* topology between mitochondrial and nuclear gene markers.

Studying the level of intraspecific variation, we found that *Cobitis* individuals shared a number of dominant haplotypes of nuclear sequence markers across the geographic distribution of the particular species (Table 2). At the level of interspecific relationships, several haplotypes were shared between *C. taenia* and *C. tanaitica* in the nuclear *28S*, *ACT-2*, *AtpB*, *N2*, *N6*, and *Rhod* loci (Table 2). At the mitochondrial *cytb* locus we obtained 23 unique species-specific haplotypes, which clustered in two main lineages that differed by 4.18% net sequence divergence: one included only *C. taenia* and second encompassed *C. elongatoides* and *C. tanaitica* (Figure 2). Deeper geographic structuring of mtDNA was found in *C. tanaitica* with two well-supported clades, the eastern clade (haplotypes H17–19; 0.93 posterior probabilities) from the Azov region and the western clade (haplotypes H8, H16–17, and H21–23; 0.99 posterior probabilities) from the Delta of Danube River and Odra River basin. The two clades differed by 0.89% net sequence divergence. *C. tanaitica* appeared paraphyletic to *C. elongatoides*: we found that *C. elongatoides* formed a sister clade to the western lineage of *C. tanaitica* with the net sequence divergence equal to 0.48%. The divergence between the eastern clade of *C. tanaitica* and *C. elongatoides* was 0.71%.

**Table 2:** Spined loaches (*Cobitis*) used in this study.

Taxon	Map No., Locality, Country	Coordinates	Locus and Haplotype									
			*28S*	*Act-2*	*AtpB*	*N2*	*N4*	*N6*	*Rag1*	*Rhod*	*RpS7*	*cytb*
***C. taenia***	1, Bolsh. Uzen' R., Kazakhstan	49.653N, 49.479E	H1	H1,H2	H1	H1	H1	H1	H1	H1	H1	H1
	2, Haaren Cr., Germany	53.083N, 7.833E	H1	H3	H1	H1	H1	H1	H2	H1	H2,H3	H2
	3, Alma R., Ukraine	44.586N, 33.619E	H1	H1	H1	H1	H1	H3	H1	H1	H1	H1
	4, Odra R., Germany	52.569N, 14.604E	H1	H2,H3	H1	H1	H1	H1	H1, H3	H1	H1,H4	H3
	5, Kodyma R., Ukraine	47.936N, 30.765E	H1	H2,H3	H1	H1	H1	H1,H2	H3	H1	H1	H4
	6, Smortawa R., Poland	50.983N, 17.367E	H1	H3	H1	H1	H1	H1	H4	H1	H1,H5	H5
	7, Malaren L., Sweden	59.276N, 17.659E	H1	H3	H1	H1	H1	H2	H1,H5	H1	H6	H1
	8, Wite Nete, Belgium	51.108N, 4.531E	H1	H3	H1	H1	H1	H1	H1	H1	H6,H7	H1
	9, Dnieper R., Russia	55.573N, 33.137E	H1	H1,H3	H1	H1	H1	H1	H1	H1	H1	H1
	10, Seine R., France	48.300N, 4.083E	H1	H3	H1	H1	H1	H1	H1	H1	H6	H1
***C. elongatoides***	11, Odra R., Poland	50.883N 17.717E	H2	H4	H2,H3	H2,H3	H2	H4	H6,H7	H2	H8	H6
	12, Tazlau, Romania	46.405N, 6.729E	H2	H4,H5	H3,H4	H6	H2,H5	H4	H6	H2	H9	H7
	13, Prut R., Romania	46.199N, 28.033E	H2	H5	H3,H5	H4	H2,H5	H4	H7	H2	H8	H8
	14, Spree R., Germany	51.363N, 14.514E	H2	H5,H6	H3	H3,H6	H2	H4	H8	H2	H8	H9
	15, Pšovka Cr., Czech Rep.	50.370N, 14.552E	H2	H5	H3	H4	H2	H4	H9	H2	H8	H10
	16, Mur R., Austria	47.262N, 14.583E	H2	H4	H2	H4	H2	H4	H8	H2	H8	H11
	17, Comana, Romania	44.168N, 26.148E	H2	H5	H3,H6	H4	H2	H4	H10	H2	H8	H12
	18, Szodrakosz Cr., Hungary	47.733N, 19.133E	H2	H5	H2	H4	H2,H5	H4	H7,H11	H2	H8	H13
	19, Kamchya R., Bulgaria	43.029N, 27.535E	H2	H5	H2,H3	H4,H5	H3	H5	H7,H11	H2	H8,H10	H14
	20, Kirčevo, Bulgaria	43.031N, 24.364E	H2	H5	H3,H4	H3	H2	H4	H7,H11	H2,H3	H8	H15
***C. tanaitica***	21, Sinoe L. , Romania	44.633N, 28.883E	H1	H7	H1	H1	H4	H1	H12	H1	H11	H16
	22, Odra R., Poland	50.883N, 17.717E	H1	H7	H1,H7	H1	H4	H1	H12	H1	H11	H17
	23, Manych R., Russia	45.991N, 43.429E	H1	H3	H1	H1	H4	H6	H13	H1	H12	H18
	24, Manych R., Russia	45.991N, 43.429E	H1	H3	H1	H1	H4	H6	H13	H1	H12	H18
	25, Kuban R., Russia	44.828N, 41.769E	H1	H3	H1	H1	H4	H6	H13	H1	H13	H18
	26, Kuban R., Russia	44.828N, 41.769E	H1	H3	H1	H1	H4	H6	H13	H1	H13	H19
	27, Kuban R., Russia	44.828N, 41.769E	H1	H3	H1	H1	H4	H7	H13	H1	H13	H20
	28, Sinoe L., Romania	44.633N, 28.883E	H1	H7	H1	H1	H4	H2	H12	H1	H11	H21
	29, Sinoe L., Romania	44.633N, 28.883E	H1	H7	H1,H7	H1	H4	H2	H12	H1	H11	H22
	30, Sinoe L., Romania	44.633N, 28.883E	H1	H7	H1	H1	H4	H2	H12	H1	H11	H23
***C. fahirae***	32, Manyas L., Turkey	40.133N, 28.049E	H3	H8	H8	H7	H6	H8	H14	H4	H14	H24
***C. vardarensis***	33, Treska R., Albania	42.000N, 21.335E	H4	H9	H9	H8	H7	H9	H15	H5	H15	H25
***C. paludica***	31, Duraton R., Spain	41.366N,−3.902W	H5	H10	H10	H9	H8	H10	H16	H6	H16	H26

### Phylogeny of the Gene Trees and the Species Tree Estimation

Nuclear data provided strong support for the sister position of *C. taenia* and *C. tanaitica*; eight of nine nuclear loci consistently supported (*C. taenia*, *C. tanaitica*) monophyly, while one locus (*Rag1*) did not resolve the relationship between the two species. No locus significantly contradicted the sister relationship between *C. taenia*, *C. tanaitica*. In contrast, the mitochondrial *cytb* gene strongly supported the sister position of (*C. elongatoides*, *C. tanaitica*), with *C. tanaitica* being paraphyletic to *C. elongatoides*. Hence, we observed strong mito-nuclear conflict that was fixed over the entire distribution range of *C. tanaitica*.

The relationships among *C. elongatoides C. fahirae*, and *C. vardarensis* appeared unresolved from patterns in gene tree topologies across mito-nuclear markers (Figures 2).

We applied the Bayesian estimation of species trees (BEST; [62]) that allows for stochastic differences of topology of individual gene trees resulting from lack of gene lineage coalescence between speciation events. BEST was run in two parallel analyses, either with nuclear markers only or in combination with mitochondrial data. Both analyses consistently identified (*C. taenia*, *C. tanaitica*) monophyly with high posterior probability (∼1, Figure S1). However, the tree topology was not fully resolved with respect to *C. elongatoides*, *C. fahirae* and *C. vardarensis* branching. *C. vardarensis* was either positioned at the basal species of the *C. sensu stricto* (nine nuclear loci data, Figure S1A), or the polytomy was present in the consensus species tree (combined nuclear and mitochondrial data, Figure S1B).

### Testing of Incomplete Lineage Sorting of *C. tanaitica* mtdna vs. Hybridization Scenarios

We employed three methodologically distinct approaches to discern whether the contrasting mito-nuclear signal may result from the retention of ancestral polymorphism or indicates some hybridization events.

#### A) Topology-Based Tests Of Mito-Nuclear Discordance

To understand a cause of mito-nuclear conflict in *C. tanaitica*, we first performed a purely theoretical test taking into account only the topological information suggesting that eight out of nine nuclear loci supported (*C. taenia*, *C. tanaitic*a) monophyly, while mtDNA indicated a ((*C. elongatoides*, *C. tanaitic*a) *C. taenia*) tree topology. Assuming that true species tree is ((*C. taenia*, *C. tanaitic*a) C. elongatoides) as indicated by BEST, we used coalescence simulation to evaluate the probability of retention of mitochondrial ancestral polymorphism as a function of the length of (*C. taenia*, *C. tanaitic*a) internode. We assumed that studied loci represent a random sample from the entire *Cobitis* genome and we simplistically modeled single sample per species. This was because Rosenberg [21] showed that with multiple samples per species, the probability of topological gene tree concordance in a three-species model is a complicated function of not only the sample numbers but also of actual population sizes and speciation times, for which we do not have independent estimates.

**Figure 3:**
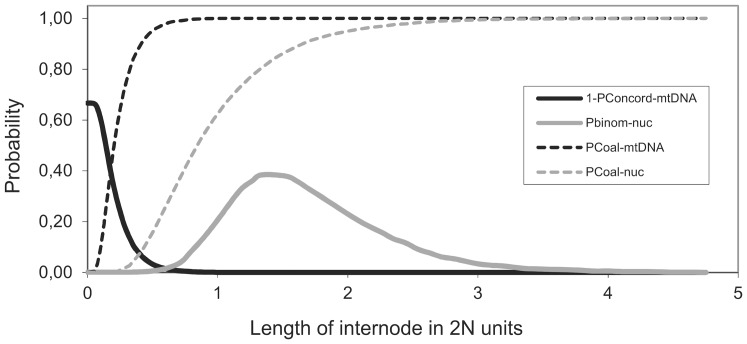
Probability densities of four parameters in coalescence simulation as functions of length of internode. Graphical visualization in which (1–P_Concord-mtDNA_) denotes the probability density of observing discordant mtDNA phylo tree; (P_binom-nuc_) denotes the probability density of observing eight topologically concordant nuclear gene trees out nine studied nuclear loci in total; (P_Coal-mtDNA_) denotes the cumulative probability of mtDNA coalescence along the internode and (P_Coal-nuc_) denotes the cumulative probability of coalescence of nuclear locus along the internode. Note that there is very small intersection of probability densities allowing for observing eight out of nine topologically concordant nuclear loci while having discordant mtDNA tree (see the text for details).

Figure 3 demonstrates that the probability of a deep coalescence pre-dating the split between C. elongatoides and (*C. taenia*, *C. tanaitica*) ancestor depends on the length of interval between the speciation events. When the interval is short, there is high probability of observing a topologically discordant mtDNA gene tree due to incomplete lineage sorting (if the interval equals zero, the probability is 2/3). In such case, however, there is a negligible chance to sample simultaneously eight out of nine nuclear loci with identical topology. On the other hand, when the internode length increases, the proportion of topologically concordant nuclear loci grows due to coalescences occurring in the (*C. taenia*, *C. tanaitica*) ancestral population, but the probability of observing a topologically discordant mtDNA gene tree rapidly decreases. Furthermore, when the internode length is high, it becomes unlikely to find any locus with discordant topology.

As apparent from the Figure 3, it is unlikely under any simulated length of the internode to observe the retention of mtDNA ancestral polymorphism when eight out of nine nuclear loci indicate identical topology. The overall probability of observed mito-nuclear conflict due to incomplete lineage sorting was negligible (*p* = 0.009).

#### B) Test Using Sequence Variability Based On Isolation Model (Speciation With No Gene Flow)

**Figure 4:**
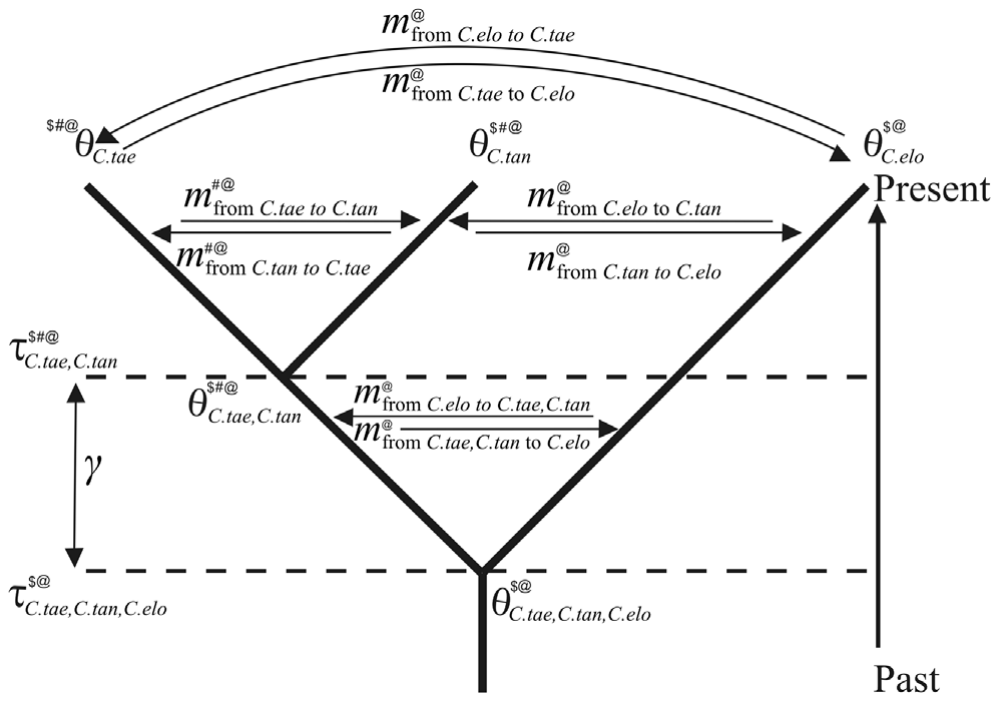
Parameters calculated from alternative tests using sequence data to explain *C. tanaitica* mito-nuclear discordance. Contemporary and ancestral population sizes are denoted by (*θ_C.tae_*, *θ_C.tan_*_,_ *θ_C.elo_*_,_ *θ_C.tae,C.tan_*, *θ_C.tae,C.tan,C.elo_*). Divergence times are denoted by (*τ_C.tae,C.tan_* and *τ_C.tae,C.tan,C.elo_*), and interval between those times is denoted by (*γ*). Migration rates are denoted by (*m*) with relevant index. All parameters are scaled by mutation rate *μ*, and can be converted to absolute values using the relations *θ* = 4*Nμ* (where *N* is effective population size), *m* = m/*μ* (where m is gene-flow rates per gene copy per generation, *τ* = t*μ* (where t is a time of population splitting at *τ* generations in the past), and *γ* = t*μ*. Parameters estimated by BPP program are denoted by ($), those by IM by (#), and those by IMa2 by (@). The parameter *γ* was calculated from *τ*s given by BPP and ds programmes. *C. taenia* (*C.tae*), *C. tanaitica* (*C. tan*), and *C. elongatoides* (*C. elo*).

In a second approach, we used nuclear sequences to estimate the population sizes and speciation times of studied species and their ancestral taxa assuming both species tree topologies provided by BEST (Figure S1). Such estimates served to evaluate the probability that the (*C. taenia*, *C. tanaitica*) internode was short enough and/or the ancestral population large enough to allow the retention of mtDNA ancestral polymorphism. Population sizes and internode lengths were estimated with the BPP [12], [63] program Version 2.2 and final parameter estimates (Table 3 and Figure 4) were substituted into equation 5 by Rosenberg [21] to calculate the probability of topological discordance of the mtDNA gene tree. We found negligible probability that the mtDNA incongruence is caused by incomplete lineage sorting under both assayed species tree topologies (*p*≤0.002).

**Table 3:** Prior and posterior distributions of parameters in the BPP Bayesian analysis of the nine nuclear loci.

Parameter	Gamma prior	Prior	Posterior
	(α, β)	Mean (95% interval)	Mean (95% interval)
*θ_C.tae_*	(2, 617)^a^	0.003240 (0.000390, 0.009030)	0.004711 (0.001715, 0.010027)
*θ_C.tan_*		0.003240 (0.000390, 0.009030)	0.002253 (0.000542, 0.006071)
*θ_C.elo_*		0.003240 (0.000390, 0.009030)	0.004988 (0.002547, 0.008695)
*θ_C.tae, C.tan_*		0.003240 (0.000390, 0.009030)	0.002799 (0.000694, 0.005922)
*θ_C.tae, C.tan, C.elo_*		0.003240 (0.000390, 0.009030)	0.003333 (0.000439, 0.008074)
*τ_C.tae, C.tan, C.elo_*	(2, 167.8)^a^	0.011920 (0.001440, 0.033200)	0.005595 (0.003016, 0.007991)
*τ_C.tae,C.tan_*	from analysis^b^		0.001573 (0.000628, 0.002936)

a Priors set with *Ne* = 225,000 covering Watterson's [91] θ estimate (*θ*_W_ = 0.002, *Ne = *138,889) and from branching event t = 3.31 Mya between European closely related *Cobitis* species [104]. Relatively fast autosomal mutation rate (*μ*) of 3.6×10^−9^ estimated in vertebrates [105] was used to transform prior expectations of *θ* and *τ* from absolute estimates of *N_e_* and t. Both *τ* and *θ* are measured as the expected number of mutations per site.

b Prior for the node age was generated from the Dirichlet distribution ([63]: equation 2).

*C. tae* = *C. taenia*, *C. tan* = *C. tanaitica*, *C. elo* = *C. elongatoides.*

#### C) Test of Gene Flow Among Spined Loaches

In a third approach, we applied an isolation with migration model using the two-population IM [64], [65] and multi-population IMa 2.0 [14], [66] programs for studying the divergence of *Cobitis* species including a parameter of gene flow (Figure 4).

**Figure 5:**
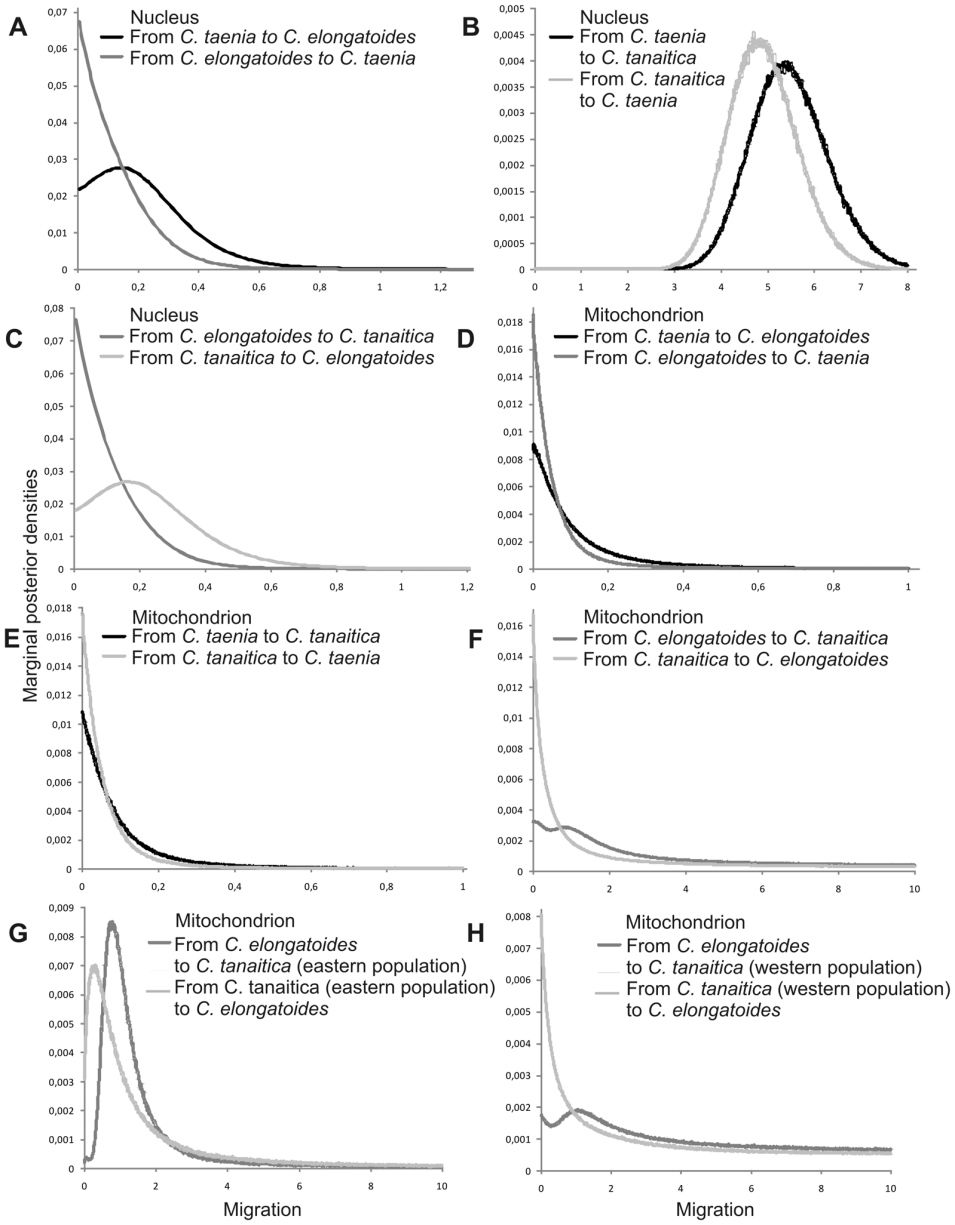
Posterior probability distributions for migration rates from two-population IM analysis. Coalescent-based estimates of migration rates (scaled by mutation rate) for three studied species inferred separately from (A–C) nuclear sequence data that included nine nuclear markers, and from (D–H) one mitochondrial marker gene.

**Figure 6:**
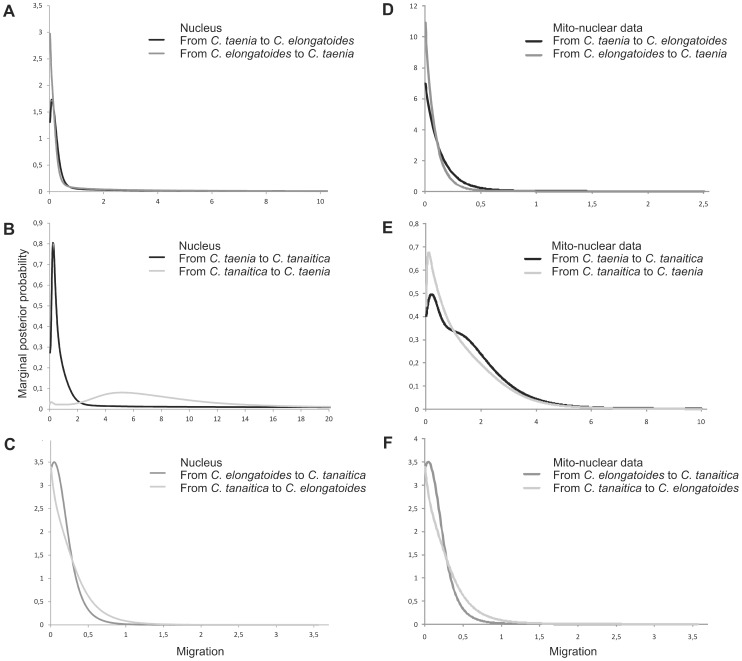
Posterior probability distributions for migration rates from three-population IMa2 analysis. Coalescent-based estimates of migration rates (scaled by mutation rate) for three studied species inferred from (A–C) nine nuclear markers and (D–F) combined mito-nuclear sequence data that included one mitochondrial marker gene and nine nuclear markers.

Both programs were applied to nuclear and mtDNA data separately and IMa2 was also applied on a combined dataset (Figures 5A–H and 6A–F). Whether or not we separated mitochondrial and nuclear data, the power to resolve migration varied under different model assumptions in IM versus IMa2, but signals suggesting migration events between species-pairs were more or less consistent (Figures 5B,F–H and 6B, E–F, Tables S1 and S2). The resulting signal for a gene flow among *Cobitis* species was mostly related to significant signatures of nuclear gene flow between *C. taenia* and *C. tanaitica*, which was supported by both IM and IMa2 programs. Consistently with this result, the investigation of the distribution of the number of migration events in the sampled gene genealogies separately for each locus found 20 migration events from *C. taenia* to *C. tanaitica* and 3 migration events for the opposite direction (combined Ima2 data), all related to nuclear loci. The inference of mtDNA gene flow was more complicated. For analysis of solely mtDNA data in the IM, we performed two types of IM runs. First, we treated all *C. tanaitica* samples as single population. Second, given its clear separation into western and eastern mtDNA phylogroup, we also analysed the gene flow between *C. elongatoides* and either eastern or western *C. tanaitica* clade to ensure the homogeneity of the study population in the model. Significant and unidirectional mitochondrial gene flow was observed only from *C. elongatoides* to the eastern *C. tanaitica*. When analyzing *C. elongatoides* and either all individuals of *C. tanaitica*, or only the western *C. tanaitica* sub-dataset, the IM provided bimodal posterior distribution for migration and for the split time (*t*) parameters. While one peak in posteriors for the migration (MLE) was always greater than zero, the second peak was located at zero (Figure S2). The program was apparently not able to distinguish between scenarios of recent split with no migration and older split with higher migration. Similarly, combined nuclear and mtDNA data in IMa2 analysis indicated possible migration from *C. elongatoides* to *C. tanaitica*, which was insignificant, however. We therefore made an investigation of the distribution of the number of migration events in the sampled gene genealogies and found 2 migration events related to the gene flow from *C. elongatoides* to *C. tanaitica* at the mtDNA locus. A detailed description of the results for the two-population IM model and three-population IMa2 model are given in Text S1.

## Discussion

### Discordance between mtDNA and Nuclear DNA IN *C. tanaitica*

*C. taenia* and *C. elongatoides* represent well-defined, non-sister taxa based on the phylogeny of both mtDNA and nuclear markers. In contrast, *C. tanaitica* clusters with *C. elongatoides* in mtDNA, while it appears as the closest relative to *C. taenia* in the nuclear genes, even sharing some haplotypes at six nuclear loci (Figure 2 and Table 2). The mito-nuclear discordance was fixed across the whole range of *C. tanaitica* (Table 2) including regions, where it occurs in distant allopatry from both other species. This finding is in agreement with previous investigations of six diagnostic species-specific allozyme loci of 58 *C. tanaitica* specimens [50].

Sharing of similar alleles by two animal taxa has been documented in various cases that differ in the geographic extent of such event and in frequency of shared haplotypes e.g. [22], [23]. Toews and Brelsford [48] review number of cases, when mito-nuclear discordance has achieved near fixation (greater than 95%). However, only in few such cases the mito-nuclear discordance has achieved 100% fixation in 100% geographic extent as in *C. tanaitica*. To our knowledge, all previously reported cases are distributed on a rather small biogeographic scale (e.g., [24]–[26], [67], while *C. tanaitica* range spans over Eastern and Central Europe (Figure 1G). *C. tanaitica* thus represents a rare example among vertebrates, where mito-nuclear discordance has been fixed on a large geographic scale including distant allopatric regions.

### Incomplete Lineage Sorting or Hybrid Introgression?

Known examples of mito-nuclear discordance, reviewed in [22], [23], [48], have often been attributed to introgressive hybridization but the presence of incomplete lineage sorting were rarely explicitly tested statistically [68]. Given that all statistical tests are necessarily based on simplifying assumptions, which may be violated in the real world, present study applied two methodologically different approaches to evaluate the role of incomplete lineage sorting and an additional approach to detect signs of a possible gene flow among species. We are aware that our conclusions may be affected by sample size and strategy because missing a basal genealogical lineage would artefactually decrease the estimates of *θ* and lead to overconfidence in the tests of incomplete lineage sorting. However, we sampled all phylogeographic lineages identified in previous studies (Figure 1G). Moreover, because all topologies of coalescent trees are equiprobable [69], the probability that additional samples would represent a new root to sampled intraspecific variability is only (2/(k*(k+1))) (*k* stands for number of sampled lineages) [70]. Assuming a more or less homogeneous population structure, our sample size of 20 chromosomes per species implies only ∼1% probability that we missed the true root, suggesting that our sampling scheme adequate for the question at hand.

Both tests of incomplete lineage sorting also relied on the choice of implemented species tree. Although the monophyly of *C. tanaitica* and *C. taenia* was strongly supported, the relevance of other possible topologies should be discussed. Species trees assuming (*C. elongatoides*, *C. tanaitica*) or (*C. elongatoides*, *C. taenia*) sister positions would imply that *C. taenia* and *C. tanaitica* have largely retained ancestral polymorphism (even sharing haplotypes at six out of nine nuclear markers), while *C. elongatoides* diverged rapidly from its putative sister species in all nuclear markers. Given that we found no evidence for rate heterogeneity in any locus, we consider these scenarios unlikely. The topology assuming a hard (*C. taenia*, *C. tanaitica*, *C. elongatoides*) polytomy may also be rejected because it predicts mosaic phylogenetic patterns in mtDNA and nuclear markers due to stochastic loss of alleles over time [71], [72], which has not been observed in *C. taenia* and *C. elongatoides.*

Altogether, both the topology-based test and the test based on isolation model rejected the possibility that *C. tanaitica* gained reciprocally monophyly to *C. elongatoides* in the nucleus while retaining the mtDNA lineage from the common ancestor. It strongly suggests that hybridization must be assumed to explain the observed mito-nuclear discordance. On the other hand, our tests do not rule out the persistence of incomplete lineage sorting among nuclear genes of *C. tanaitica* and *C. taenia*, because discriminating between introgression and incomplete lineage sorting is notoriously difficult when loci have not had enough time to diverge [40]. Despite this, we show in concordance with previous multilocus studies [9], [73] that even a small number of loci can be used to effectively detect hybridization.

Results from IM and IMa2 provided some general suggestions about possible directions of gene flow among species. While nuclear gene flow between *C. tanaitica* and *C. taenia* was significant, potential mtDNA gene flow from *C. elongatoides* to *C. tanaitica* (Figure 5B and 6B) was indicated by nonzero peaks (Figure 5F,G and H) as well as by inferred mtDNA migration events, which were, nonetheless, mostly insignificant.

We are aware that the failure to reveal significant traces of migration might have been caused by some limiting assumptions of IM/IMa2 analyses that are not fully compatible with our population dataset (IM may be compromised by the existence of ‘ghost populations’ [74]; IMa2 relies on a defined species tree and a high number of loci, as the more populations and parameters require more data to obtain useful parameter estimates [14]; Both methods are appropriate for the analysis of recently separated populations with ongoing gene flow [65], so a greater phylogenetic distance between (*C. taenia*, *C. tanaitica*) clade and *C. elongatoides* might have caused problems of recovering signal of older mitochondrial introgression events (see further in the text); Also, IM considers only two populations, leaving us with two options how to treat apparent phylogeographic structuring (western and eastern) of *C. tanaitica* mtDNA: (1) keeping the both sub-populations as a single population, which violates the assumption of panmixia, or (2) treating each population in separate analyses, which violates the assumption of no gene flow with other populations). Together, these circumstances might have caused analytical difficulties for IM in recovering signal of migration from *C. elongatoides* to *C. tanaitica* treated as either single population, or considering only its western clade (Figures 2, S1, 5F and 5H). Bimodal posterior distributions for migration and split time (*t*) parameters (Figure S2), most likely reflected two attractors (J. Hey, pers. comm.), suggesting either the scenario of recent population split between *C. elongatoides* and *C. tanaitica* with zero migration into *C. tanaitica*, or older split with strong migration into *C. tanaitica*. We therefore consider IM and IMa2 results only as supplementary analysis in order to get a basic idea about directions of gene flow.

### Origin of *c. tanaitica* Mosaicism

The consensus of all applied methods suggests that mito-nuclear genomic mosaicism of *C. tanaitica* may not be explained by the retention of ancestral polymorphism, but most likely resulted from interspecific gene flow, which concerned nuclear gene flow between *C. taenia* and *C. tanaitica*, and a parallel mtDNA transfer likely from *C. elongatoides*.

How could such introgression occur over the whole range in *C. tanaitica*? Available evidence suggests that gene flow among spined loaches is a rather old episode. Recent or ongoing introgressions, either nuclear or mitochondrial, seem unlikely, because necessary drivers of the gene flow (i.e. non-clonally reproducing hybrids) are absent, or so rare that they remain undetected despite extensive field sampling and laboratory experiments [50], [53]–[56]. The contemporary hybrid forms appear as fixed heterozygotes and are either infertile (rare hybrid males [50], [55], or display strictly clonal heredity [50], [53], [55], [57], [59]. The hybrid nature has been in proven by experimental crossings in *C. elongatoides-taenia* females and evidenced by population genetic methods also in hybrids possessing *C. tanaitica* genome [53], [54]. It is also noteworthy that hybrids between *C. taenia* (2n = 48 chromosomes; Figure 1A–B) and *C. tanaitica* (2n = 50 chromosomes; Figure 1C–D), yet undetected in nature, would possess an odd chromosome number (2n = 49) probably causing defective meiosis.

Further support for the hypothesis of ancient gene flow between *C. tanaitica* and other two species comes from the fact that the mtDNA variation of contemporary *C. elongatoides*-*tanaitica* clonal hybrids is fully embedded within the *C. elongatoides* mtDNA cluster (e.g., [57]), while *C. tanaitica's* mitochondrion forms distinct clusters. Therefore, the mtDNA of *C. tanaitica* was probably inherited from *C. elongatoides*-like ancestor before Pleistocene/Holocene origin of contemporary clonal hybrids. Given that the contemporary range overlap between spined loach species is very limited (Figure 1G) and that *C. elongatoides* has not recently expanded to the Azov and Kuban regions [57], such introgression must have happened long enough ago to allow mtDNA divergence between western and eastern populations of *C. tanaitica*. Similarly, the intensive nuclear gene flow between *C. taenia* (2n = 48) and *C. tanaitica* (2n = 50) suggested by the IM and Ima2 models must have occurred before chromosomal rearrangements had taken place between the two species.

Ideally, the hypothesis of rather older gene flow should be complemented by the estimates of times when inferred migration events took place and when species diverged [75]. However, we refrained from this approach for two reasons. First, we did not obtain reliable estimates of *t* and *θ*_A_ in some IM runs (Tables S1 and S2) suggesting that the data do not contain enough information for rigorous estimates of divergence times. Second, existing methods for estimating the number and times of migration events in separate loci [76] are limited by the fact that genealogies with different migration timing can have similar posterior probability [77]. The application of migration time estimates into studies of reticulate speciation is thus non-trivial, especially when gene flow has varied through time [76]–[78]. Instead, we combined biologically relevant data on chromosomal and genetic variability, phylogeography and reproductive modes of spined loach species and their hybrids to show that hybridization among the three species is an ongoing process (e.g., [53], [55]) but any genomic replacements or introgressions into *C. tanaitica* most likely pre-dated the origin of contemporary clonal hybrids.

Identifying the proximate mechanisms of hybrid introgression and a type of hybrid mediators is more difficult. To understand the causal link between interspecific hybridization and asexual reproductive mode of hybrids, Moritz et al. formulated the “balance hypothesis” [79], which predicts that hybrids between closely related species usually retain meiosis. The growing divergence between parental species increases the proportion of hybrids with aberrant meiosis due to incompatibilities in the meiosis-regulating genes. This process may continue until eventually the F1 hybrids produce entirely clonal gametes [79]. The balance hypothesis offers a possibility that fertile hybrids with Mendelian inheritance, which are virtually absent in the present, have been common in the past when the divergence between *C. tanaitica* and *C. elongatoides* was lower. Such hybrids might have mediated directed backcrossing to sexual species in *Cobitis*.

Alternatively, the transfer of a mitochondrial and/or complete nuclear genome might have been mediated by hybrids with non-Mendelian heredity, but not completely clonal, e.g. with hybridogenetic reproduction. Hybridogenetic hybrids are sperm-dependent parthenogens with hemiclonal gametogenesis that combine two genomes, e.g., one of species *A* and the other from species *B*. They usually discard the complete genome of one species (e.g. *A*) and clonally transmitting second parental genome (*B*). Mating with the species *A* restores the hybrid constitution of the progeny. However, there are cases of diploid *AB^A^* or triploid *ABB^A^* biotypes (hybrids with *^A^*-type mitochondrion) producing haploid *B^A^* gametes [33]–[38], [80], [81]. When such gametes are fertilized by a *B*-sperm, they give rise to mito-nuclear mosaic *BB^A^* genome with normal Mendelian segregation (e.g., [37], [38], [82], [83]). Therefore, such a mechanism of saltational evolution may easily supersede many unidirectional backcrosses to a sexual population through hybrids with Mendelian inheritance, and result in a homogeneous nuclear genome derived from only one parental species.

There is no indication of hybridogenesis in currently occurring European spined loaches despite intensive crossing experiments and oocyte electrophoreses. However, distantly related loaches from eastern Asian have the capability of hybridogenetic reproduction [37]. A simultaneous occurrence of clonal and non-clonal hybrid reproduction in some animal complexes, e.g. *Ambystoma* [46], *Squalius* [33], [80], suggest that more than one type of reproduction may occur at least at some point in evolution of these animal systems. It is interesting in this context that published data on life-bearing hybrid fish *Poeciliopsis* demonstrate that the switch from hybridogenesis to gynogenesis may be induced by polyploidization (2n hybrids are hemiclonal, while their 3n descendants are gynogenetic [84]. By analogy, it is possible that contemporary *C. elongatoides-tanaitica* hybrids, which occur as triploids, might have originated from diploid hybrid ancestors with different reproductive mode. When considering this possibility in future research, it should be kept in mind, however, that both actual di- and triploid hybrids between *C. elongatoides* and *C. taenia* species-pair are gynogenetic [50], [53]–[55], [57]–[59].

## Conclusions

Studies demonstrating secondary displacement of original markers across whole-ranges of allopatrically distributed species (see reviews in [5], [22]) are usually complemented by evidence of hybrids with Mendelian heredity. Hybrid systems displaying non-sexual reproduction have traditionally been considered as an effective barrier to genomic introgression. Recently this view is changing (e.g., [85], [86]).

Putting the data from European spined loaches together, the most parsimonious explanation of the observed mito-nuclear discordance suggest that hybrid drivers mediating interspecific gene flow through non-clonal reproduction have been common in the past but vanished as parental taxa diverged and clonally reproducing hybrids began to appear. If so, spined loaches would constitute one of the strongest support of the “balance hypothesis” [79]. Although we were unable to distinguish, whether proximate drivers of introgression into C. tanaitica were hybrids with Mendelian inheritance or hybrids with inheritance not entirely clonal, e.g. [85], [86], our study demonstrates that massive introgression may be detected even in the apparent dominance of clonal hybrids in contemporary populations - an important finding in its own right that extends our knowledge about the stability of reproductive barriers and reproductive modes in animals.

The reticulate evolution of the *C. taenia* hybrid complex demonstrates that evolutionary histories of organismal groups combining sexual and non-sexual reproductive modes may be complex. It follows that analytical tools to reconstruct their histories should be adequately complex. These should combine fine-scale geographical sampling and crossing experiments with modern analytical tools allowing more complex models, which assume that gene flow between diverging populations may vary in time, or even among genomic regions.

## Materials and Methods

### Ethics Statement

‘Valid Animal Use Protocols’ CZ 00221 issued by Ministry of Agriculture of the Czech Republic (CR) were in force at IAPG AS CR, Liběchov. The DNA material preserved in 96% ethanol (a piece of fin clipped from the specimens) was obtained by exchange between collaborating teams between 1989 and 2009. Where applicable, fishing and tissue collection were carried out with appropriate permissions from local authorities to the external collaborators. The photos of spined loaches were taken under the permissions of the Polish Government No. DLOPiK-op/ogiz-4200/V-13/4443/06/aj, DLOPiK-op/ogiz-4200/V-11/7656, 9940/07/08/łw and No. DOPozgiz-4200/V-27/1612/09ls. Fish for photographing were handled by LC who has Certificate of competency according to §17 of the CR Act No. 246/1992 coll. on the Protection of Animals against Cruelty (Reg. no: CZU 955/06), provided by Central Commission for Animal Welfare, which authorizes animal experiments in the CR.

### Sample and Data Collection

Spined loaches were sampled from 33 localities across the species geographical range. To consider hybridization and incomplete lineage sorting in a phylogenetic framework, several individuals are sampled from each species of interest [9], [30], [73]. We therefore used ten individuals each of *C. taenia*, *C. tanaitica*, and *C. elongatoides* to cover complete geographic variability for the sequencing analyses (Figure 1G; Table 2). In addition, we included one specimen from two non-Mediterranean *C. sensu stricto* species that are not involved in hybridization but belong to the same phylogenetic clade, namely *C. fahirae* and *C. vardarensis*. A single individual Iberian spined loach, *C. paludica*, was used as the outgroup in the phylogenetic reconstructions (Figure 2).

Because different regions of the genome are expected to coalesce and introgress at different rates due to the action of selection and drift and because mtDNA is only maternally inherited compared to the common biparental heredity of the nucleus, we sampled genetic data from across the genome. We sequenced the mitochondrial cytochrome *b* gene (c*ytb*) together with nine nuclear loci: the ribosomal RNA gene (*28S*); parts of the coding regions of the recombination activating (*Rag1*) and rhodopsin (*Rhod*) genes; intron 2 and partial cds of the actin gene (*Act-2*); intron and partial cds of the adenosine triphosphate synthase beta subunit gene (*Atp-B*); intron of the S7 ribosomal protein gene (*RpS7*); three anonymous noncoding genomic DNA fragments (abbreviated as *N2*, *N4*, and *N6*); see Table 2 and Tables S3, S4, and S5 for details of laboratory procedures; Table S6 summarizes GenBank accessions both for sequences corresponding with the previously published haplotypes, or new haplotypes.

### DNA Sequence Polymorphism

Sequences were aligned in ClustalW [87], and manually edited in BioEdit 7.0.9.0 [88] and were then manually checked and edited. All nuclear sequence traces were inspected visually and haplotypes of heterozygote individuals were resolved using diploid homozygote sequence traces [89]. The amino acid translation of the coding sequences was examined for stop codons. We calculated haplotype diversity, nucleotide diversities [90], *θ* [91], net sequence divergence between populations, and the number of replacement versus synonymous mutations using DnaSP 5.0 [92].

### Assessment of Recombination and Selection

We evaluated the prevalence of historical recombination within gene sequences using the four-gamete test [93]. For all subsequent analyses that assumed no within-locus recombination, we discarded the sites left or right of the putative recombination events to retain the longest possible contiguous non-recombined sequence.

Because locus-specific selection might invalidated the inferences of applied analyses, we tested for the presence and type of locus-specific selection using the Hudson-Kreitman-Aguade test [94], (available at http://lifesci.rutgers.edu/~heylab), and compared the data against 10,000 neutral coalescent simulations assuming correlation between the number of polymorphisms and interspecific divergence at all loci. We further used the *Z* summary statistic [95] to compare the ratios of fixed replacement (FR) and fixed synonymous (FS) mutations to polymorphic replacement (PR) and polymorphic synonymous (PS) mutations, such as *Z* = log_10_((FR/FS)/(PR/PS)). *Z* was expected to be negative under purifying selection, and the significance of deviations from neutrality was tested using a 2-by-2 contingency table [96].

### Phylogeny of the Gene Trees and the Species Tree Estimation

ModelTest 3.7 [97] using the Akaike information criterion was used to select models of sequence evolution for individual loci. Under the parameters of the best-fit model, we constructed Bayesian gene trees using MrBayes 3.1 [98]. Each gene was analyzed separately using 10^7^ generations, sampling every 100 trees and using two parallel runs each with four chains (one cold and three heated; the default temperature for the chains was fixed). The final tree with posterior probabilities of each bipartition was constructed by discarding 40% of the sampled trees as burn-in. Finally, we compared all species pairs of *C. elongatoides*, *C. taenia*, *C. tanaitica*, *C. fahirae* and *C. vardarensis* to the outgroup (*C. paludica*) in MEGA 4.0 [99] and used the relative rate test to identify the rate heterogeneity of all loci [100]. Trees were visualized with the Treeview 1.6.6. program (downloaded from taxonomy.zoology.gla.ac.uk/rod/treeview.html).

The impact of various evolutionary forces may cause that gene phylogenies differ from the overall species phylogeny, which represents the evolutionary relationships of the organism as a whole [30]. We used the BEST software [62], which evaluates the most probable gene trees and gives the set of possible species trees, allowing for stochastic differences of individual gene trees resulting from coalescence in ancestral populations. We analysed two data sets (nine nuclear loci and combined nuclear and mitochondrial data), each by two parallel runs with four chains for 80 million generations and sampled every 1000 trees. We used independent gamma distributions as the prior of *θ*, setting the effective population sizes of uniparentally inherited and haploid mtDNA loci as one fourth that of autosomal markers following [101]. The stability of posterior probabilities for individual clades were analysed during the BEST runs. The trees then obtained were summarised in MrBayes software using the ‘sumt’ command. The burnin was always set to 10 million generations. The trees were viewed in the FigTree v1.4.0 (downloaded from http://tree.bio.ed.ac.uk).

### Testing of Incomplete Lineage Sorting of *C. tanaitica* mtdna vs. Hybridization Scenarios

#### A) Topology-Based Tests of Mito-Nuclear Discordance

Because we observed that eight out of nine nuclear loci were topologically concordant with the reconstructed species tree ((T,N),E), while mtDNA suggested an alternative topology, we used coalescent simulation to evaluate the probability of observed mito-nuclear conflict. This test relied on following rationale. Given the true species tree, e.g., ((A,B),C) and a single sample per species, there would be approximately 1/3 of the loci topologically concordant with the species tree if the length of the internode was close to zero [70]. The probability of encountering a topologically concordant nuclear locus (*P_Concord-nuc_*) would rise with internode length due to the occurrence of interspecific coalescences [21]. On the other hand, the probability of observing topologically discordant mtDNA gene tree would decrease faster due to its four times smaller effective population size. The task is to evaluate the likelihood of finding topologically discordant mtDNA gene tree given that 8 out of 9 nuclear loci support an alternative topology.

We used Mesquite software [102] to simulate large number of coalescence histories (10^4^) representing independent nuclear loci of the *Cobitis* genome from which we randomly sampled nine for the present study. Each simulation modeled a population of N diploid individuals (i.e. 2N gene copies) corresponding to the (A, B) internode of a hypothetical species tree with three tips. We noted, per generation, the proportion of loci where all gene copies coalesced to a single MRCA (i.e. the most recent common ancestor of all 2N copies) and calculated the cumulative probability of coalescence of nuclear locus along the internode (*P_Coal-nuc_*). When the internode is of length 0, *P_Concord-nuc_* equals ⅓ but as the internode length increases, *P_Coal-nuc_* growths and enlarges *P_Concord-nuc_* with the coefficient ^2^/_3_ (i.e., *P_Concord-nuc_* does not change when the coalescence occurs in the one third of loci that were initially concordant). Hence, we might evaluate the *P_Concord-nuc_* per generation as (⅓+⅔×*P_Coal-nuc_*). Therefore, the probability of observing eight topologically concordant loci out of nine loci studied in total is binomially distributed (hereafter denoted as *P_binom-nuc_*) and depends on the proportion of topologically concordant loci - *P_Concord-nuc_*.

Subsequently, we performed an analogous simulation for mtDNA locus with N/4 gene copies assuming a four-fold smaller effective population size for mtDNA, and calculated the per-generation probability of observing a concordant mtDNA genetic tree (*P_Concord-mtDNA_*) (Figure 3). Finally, we determined the probability of observed mitonuclear discordance as the sum over all generations of the product (*P_binom-nuc_*×(1−*P_Concord-mtDNA_*)).

The simulations were run with N = 1000 and N = 10,000 to check the stability of the result and the internode length was expressed in coalescence-time units (t/2N). Present simulation exactly holds only when assuming single sample per species but we found this simplification useful for the purely theoretical approach because *C. taenia* and *C. tanaitica* appear monophyletic relative to *C. elongatoides* in eight out of nine nuclear loci (in contrast to mtDNA). Moreover, with more samples per species, calculating the probability of topological gene tree concordance requires the knowledge of actual population sizes and speciation times, which are not known *a priori* and must be estimated from the same data as the gene trees.

#### B) Test Using Sequence Variability Based on Isolation Model (Speciation with No Gene Flow)

Before hybridization can be accepted as a reasonable explanation for the evolution of the data, incomplete lineage sorting as an alternative cause for the incongruence among gene trees and species tree has to be eliminated [9]. We adapted the approach of Joly et al. [9] for our multilocus data showing apparent mito-nuclear conflict in *C. tanaitica*. Assuming ((*C. taenia*, *C. tanaitica*,) *C. elongatoides*) species tree we tested whether mtDNA of *C. tanaitica* might be retained from the common ancestor of *C. tanaitica* and *C. elongatoides*.

We first used nuclear loci to estimate ancestral population sizes, *θ*_C. taenia, C. elongatoides, C. tanaitica_ and *θ*_C. taenia, C. tanaitica_ (*θ* = 4*N_e_μ*; where *μ* was the mutation rate in substitutions/site/generation), and species divergence times *τ*_. C. taenia, C. elongatoides, C. tanaitica_ and *τ*_. C. taenia, C. tanaitica_ (*τ = *t*μ*; Figure 4) with the Bayesian Markov chain Monte Carlo (MCMC) algorithms implemented in BPP Version 2.2 [12], [63]. This method accommodates the species phylogeny as well as lineage sorting due to ancestral polymorphism and allows for the incorporation of relative mutation rates among loci. We analyzed either data from three (*C. taenia*, *C. elongatoides*, *C. tanaitica*) or all five (*C. taenia*, *C. elongatoides*, *C. tanaitica*, *C. fahirae*, *C. vardarensis*) spined loach species with fixed (*C. taenia*, *C. tanaitica*) species tree monophyly. A broad γ prior *G*(2, 617) similar to [2], [103] was used on the population size parameters (*θ*s), covering the highest Watterson's [91] *θ* estimate (*θ*_W_ = 0.002) for populations in the study. The age of the root in the species tree (*t*0) was assigned the γ prior *G*(2, 167), while the other divergence time parameters were assigned the Dirichlet prior [63]; equation 2). The γ prior for t was calculated from the branching event (3.31 Mya) between closely related European *Cobitis* species [104]. We assumed an autosomal mutation rate (μ) of 3.6×10^−9^ estimated in vertebrates [105]. Each analysis was run three times to confirm consistency. Posterior distributions of all parameters were generated by the ds program in the BPP package. The program outputs summary statistics and the histogram indicating the probability with which any parameter estimate occurs within a specified bin of values. We calculated the internode length, (*γ*; Figure 4), by subtracting all possible combinations of *τ*_ C. taenia, C. tanaitica_ and *τ*_C. taenia, C. tanaitica, C. elongatoides_ bins weighted by the corresponding posterior probabilities.

We subsequently tested the probability of mtDNA discordance in *C. tanaitica* due to incomplete lineage sorting by substituting the estimated ancestral population parameters into equation 5 by Rosenberg [21]. The author showed that the probability of topological discordance of a gene tree may be calculated using the number of samples in each contemporary species and the ancestral population parameter, *T*, referring to the duration of phases between speciation events expressed in coalescence units (in diploids *T* = t/2*N_e_*, where “t” is the time in generations and *N_e_* is the effective haploid population size). In our case, there were two such phases, i.e., *T*_C. taenia, C. tanaitica, C. elongatoides_ _- C. taenia, C. tanaitica_ and *T*_C. taenia, C. tanaitica_. Given that *θ* and *γ* are both scaled by the mutation rate, which can be canceled out, and that *θ* is estimated from nuclear DNA, which has four time larger effective population size compared to mtDNA, both parameters may be substituted into Rosenberg's equation 5 in the place of *T*, i.e., *T*_C. taenia, C. tanaitica, C. elongatoides_ _- C. taenia, C. tanaitica_ = *γ*/0.125*θ*_C. taenia, C. tanaitica, C. elongatoides_ and *T*_C. taenia, C. tanaitica_ = *t*_C. taenia, C. tanaitica_/0.125*θ*_ C. taenia, C. tanaitica_ to calculate the probability of mtDNA topological discordance. To be conservative, we used the larger value of ancestral *θ* (*θ*_C. taenia, C. tanaitica, C. elongatoides_). To obtain the total probability of mtDNA discordance given the observed posterior distributions, we summed the discordance probabilities of all combinations of ancestral *θ* and *γ* bins weighted by their posterior probabilities.

#### C) Test of Gene Flow Among Spined Loaches

The two-population IM [64], [65] and multi-population IMa 2.0 [14], [66] programs were used to evaluate the level of gene flow among *C. taenia*, *C. elongatides*, and *C. tanaitica*. The models assume dichotomous splits of ancestral populations *t* generations ago; since then, descendant populations may or may not continue with a gene exchange (Figure 4). We found important to analyze mtDNA and nuclear loci separately because distinct genomic regions may have different histories [22], [23], especially in hybrid systems with reproductive strategies other than sexual reproduction [46], [81].

We ran the IM for *C. taenia* - *C. elongatoides*, *C. taenia* – *C. tanaitica*, and *C. elongatoides* - *C. tanaitica* species pairs first, for mtDNA sequences only and second, for nine nuclear loci. Due to the geographic structure of *C. tanaitica* for mtDNA (Figure 2), we further ran the IM analysis for two *C. tanaitica* mtDNA subsamples (the eastern and western clade) to approximate single reproductive units. We ran IMa2 with a data from three species together, using first, mtDNA locus, second, nuclear data and third, combined dataset with mtDNA and nuclear data to maximize the number of loci. We included the known ((*C. taenia*, *C. tanaitica*) *C. elongatoides*) species tree into the model. Based on the Akaike information criterion, we applied the Hasegawa-Kishino-Yano mutation model for all but the *Atp-B* and *N6* loci, where the infinite site mutation model was used. Each locus was assigned an inheritance scalar (i.e., modifier of effective population size: 1.0 for nuclear genes, 0.25 for mtDNA locus). We used uniform prior distributions of parameter ranges (IM) or calculated values for the upper bounds on prior distributions according to program Documentations, and then ran MCMC simulations. We checked that the posterior distributions fell completely within the bounds of the prior distribution. If distinct peaks and/or flat tails were observed in IM, we defined the upper bounds based on the results of previous runs, assuming that the ancestral population was not greater than the combined size of two daughter populations and taking into account the upper 95% highest posterior distribution for a given parameter. We performed several independent runs of up to eight chains (IM) and up to 80 chains (IMa2) under the Metropolis coupling with a geometric heating model to improve mixing. Each chain was initiated with a burn-in period of 100,000 updates. The total length of each analysis was at least 30 million (IM) and seven million (IMa2) updates. The final runs were repeated three times with different random seed numbers. The analyses were considered to have converged on a stationary distribution if the independent runs generated similar posterior distributions. To test whether the estimated migration rate is significantly different from zero, we used the LRT test to compare the differences between likelihoods of zero migration and the best migration rate estimates.

## Supporting Information

Figure S1 BEST analyses trees. Bayesian species trees (BEST) calculated from data based on (A) nine nuclear loci and (B) combined data set of nine nuclear loci and one mitochondrial marker gene.
(TIF)

Figure S2 Posterior probability distributions for divergence times from two-population IM analysis. Coalescent-based estimates for divergence times (scaled by mutation rate) for a split between *C. elongatoides* and *C. tanaitica* inferred from mitochondrial marker gene. (A) *C. elongatoides* and *C. tanaitica* (all individuals), (B) *C. elongatoides* and *C. tanaitica* (eastern clade), (B) *C. elongatoides* and *C. tanaitica* (western clade).
(TIF)

Table S1 Estimates of parameters from two-population IM analysis.
(DOC)

Table S2 Estimates of parameters from three-population IMa2 analysis.
(DOC)

Table S3 Protocols used for gene amplifications.
(DOC)

Table S4 Primers used in this study.
(DOC)

Table S5 PCR profiles used for gene amplifications in this study.
(DOC)

Table S6 GenBank accession numbers of spined loach (*Cobitis*) sequence haplotypes aligned in this study.
(XLSX)

Text S1 Detailed description of results from IM and IMA2 analyses.
(DOC)
