# Supplementary figures and images for: Distinguishing between Incomplete Lineage Sorting and Genomic Introgressions: Complete Fixation of Allospecific Mitochondrial DNA in a Sexually Reproducing Fish (Cobitis; Teleostei), despite Clonal Reproduction of Hybrids
Source: PLoS One. 2014 Jun 27;9(6):e80641. doi: 10.1371/journal.pone.0080641 (PMC4074047; doi:10.1371/journal.pone.0080641)

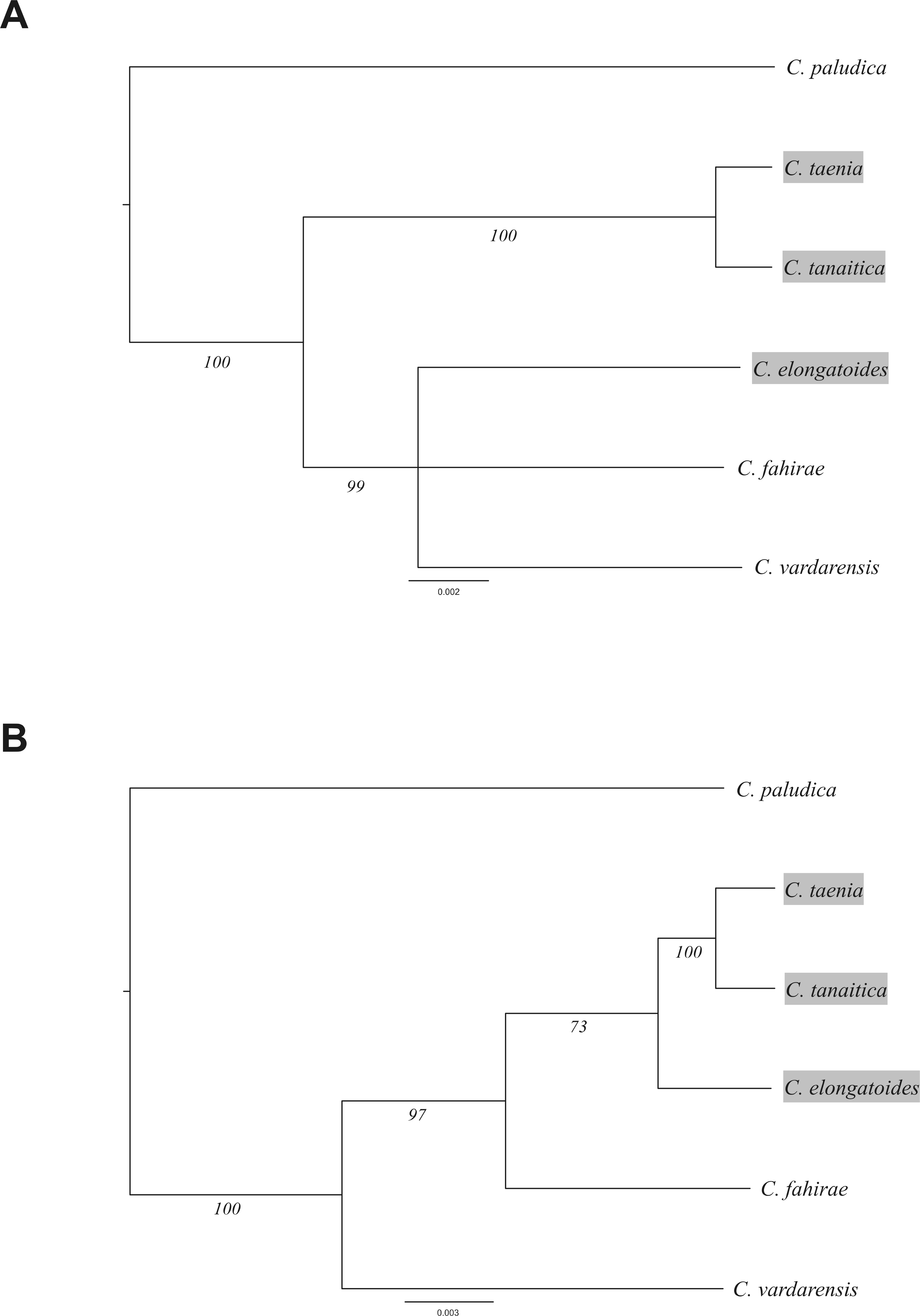

Supplement: Figure S1 — BEST analyses trees. Bayesian species trees (BEST) calculated from data based on (A) nine nuclear loci and (B) combined data set of nine nuclear loci and one mitochondrial marker gene. (TIF) [file pone.0080641.s001.tif]

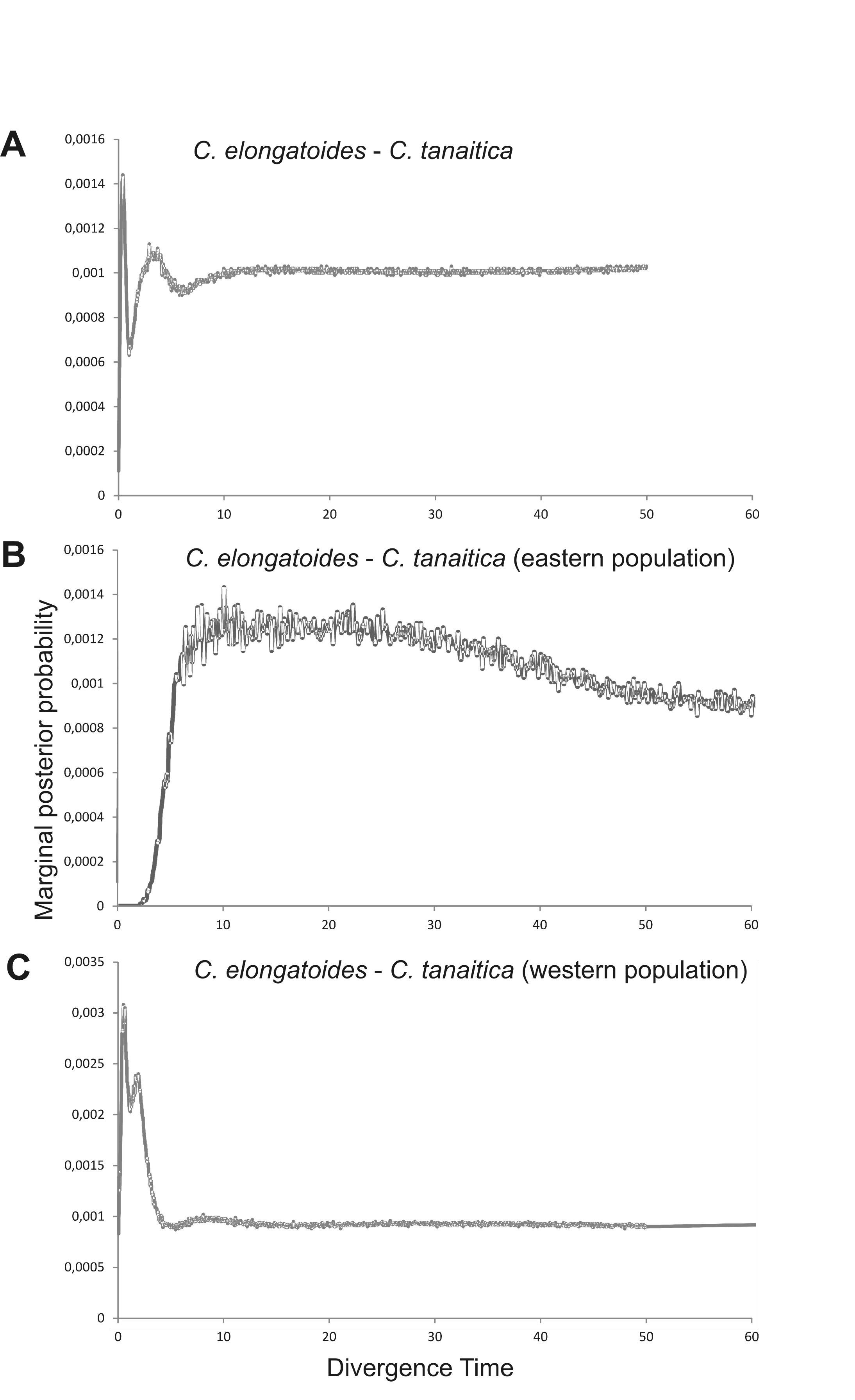

Supplement: Figure S2 — Posterior probability distributions for divergence times from two-population IM analysis. Coalescent-based estimates for divergence times (scaled by mutation rate) for a split between C. elongatoides and C. tanaitica inferred from mitochondrial marker gene. (A) C. elongatoides and C. tanaitica (all individuals), (B) C. elongatoides and C. tanaitica (eastern clade), (B) C. elongatoides and C. tanaitica (western clade). (TIF) [file pone.0080641.s002.tif]
