# Supplementary material for: Distinguishing between Incomplete Lineage Sorting and Genomic Introgressions: Complete Fixation of Allospecific Mitochondrial DNA in a Sexually Reproducing Fish (Cobitis; Teleostei), despite Clonal Reproduction of Hybrids
Source: PLoS One. 2014 Jun 27;9(6):e80641. doi: 10.1371/journal.pone.0080641 (PMC4074047; doi:10.1371/journal.pone.0080641)
Supplement: Table S1 — Estimates of parameters from two-population IM analysis. (DOC) [file pone.0080641.s003.doc]

Table S1. Estimates of parameters from two-population IM analysis. Note that *θ* (theta), *τ* (time since ancestral population splitting), and *m* (migration rate) are parameter estimates scaled by per gene mutation rate *μ* which is for nucleus equal to the geometric mean of the mutation rates of all loci. T, *C. taenia*; E, *C. elongatoides*; N, *C. tanaitica*. Nucleus includes all nine nuclear loci. Mitochondrion includes one mtDNA locus. The *m*-values on significant paths given by LRT are shown in bold. † indicates that HPD interval did not appear to be contiguous and the estimates are not reliable. MLE, Maximum Likelihood Estimates; HPD90Lo, the lower bound of the estimated 90% highest density interval; HPD90Hi - the upper bound of the estimated 90% highest density interval.

|  | | | | | |  |
| --- | --- | --- | --- | --- | --- | --- |
| Dataset | *θ*_T_ | *θ*_E_ | *θ*_TE_ | *τ* | *m*_from T to E_ | *m*_from E to T_ |
| MLE Nucleus | 0.152 | 0.333 | 4.156 | 2.575 | 0.155 | 0.005 |
| HPD90Lo | 0.059 | 0.163 | 0.021^†^ | 0.895^†^ | 0.005 | 0.005 |
| HPD90Hi | 0.311 | 0.575 | 12.624^†^ | 9.995^†^ | 0.425 | 0.275 |
| MLE Mitochondrion | 11.739 | 42.520 | 107.040 | 25.225 | 0.002 | 0.001 |
| HPD90Lo | 4.044 | 18.843 | 23.775^†^ | 4.625^†^ | 0.000 | 0.000 |
| HPD90Hi | 33.443 | 101.318 | 197.210^†^ | 49.975^†^ | 0.336 | 0.169 |
| Dataset | *θ*_T_ | *θ*_N_ | *θ*_TN_ | *τ* | *m*_from T to N_ | *m*_from N to T_ |
| MLE Nucleus | 0.336 | 0.370 | 2.349 | 1.767 | **5.380** | **4.700** |
| HPD90Lo | 0.284 | 0.300 | 0.001^†^ | 1.712 | 4.116 | 3.692 |
| HPD90Hi | 0.406 | 0.436 | 3.476^†^ | 1.812 | 6.788 | 6.100 |
| MLE Mitochondrion | 12.145 | 30.320 | 97.777^†^ | 25.975 | 0.000 | 0.000 |
| HPD90Lo | 4.281 | 12.844 | 15.640^†^ | 9.525^†^ | 0.000 | 0.000 |
| HPD90Hi | 33.990 | 72.612 | 174.671^†^ | 49.975^†^ | 0.268 | 0.158 |
| Dataset | *θ*_E_ | *θ*_N_ | *θ*_EN_ | *τ* | *m*_from E to N_ | *m*_from N to E_ |
| MLE Nucleus | 0.369 | 0.137 | 4.208 | 3.285 | 0.005 | 0.165 |
| HPD90Lo | 0.181 | 0.045 | 0.020^†^ | 1.045^†^ | 0.005 | 0.005 |
| HPD90Hi | 0.610 | 0.281 | 11.740^†^ | 9.985^†^ | 0.255 | 0.455 |
| MLE Mitochondrion | 36.044 | 19.005 | 5.024 | 0.425 | 0.045 | 0.005 |
| HPD90Lo | 11.140^†^ | 1.529 | 0.218^†^ | 0.225^†^ | 0.005^†^ | 0.005^†^ |
| HPD90Hi | 436.679^†^ | 142.210 | 436.679^†^ | 49.975^†^ | 7.645^†^ | 6.765^†^ |
| Dataset | *θ*_E_ | *θ*_N Azov_ | *θ*_EN Azov_ | *τ* | *m*_from E to N Azov_ | *m*_from N Azov to E_ |
| MLE Mitochondrion | 28.048 | 1.476 | 2826.948 | 10.150 | **0.785** | 0.315 |
| HPD90Lo | 7.381^†^ | 1.476 | 1.476^†^ | 5.450^†^ | 0.195 | 0.005^†^ |
| HPD90Hi | 2950.950^†^ | 28.048 | 2950.950^†^ | 99.950^†^ | 3.085 | 4.225^†^ |
| Dataset | *θ*_E_ | *θ*_N Delta_ | *θ*_EN Delta_ | *τ* | *m*_from E to N Delta_ | *m*_from N Delta to E_ |
| MLE Mitochondrion | 28.586 | 15.392 | 15.392 | 0.525 | 1.065 | 0.005 |
| HPD90Lo | 7.796^†^ | 0.999^†^ | 0.199^†^ | 0.075^†^ | 0.005^†^ | 0.005^†^ |
| HPD90Hi | 399.607^†^ | 386.014^†^ | 399.607^†^ | 48.875^†^ | 8.695^†^ | 8.585^†^ |
