# Supplementary material for: Distinguishing between Incomplete Lineage Sorting and Genomic Introgressions: Complete Fixation of Allospecific Mitochondrial DNA in a Sexually Reproducing Fish (Cobitis; Teleostei), despite Clonal Reproduction of Hybrids
Source: PLoS One. 2014 Jun 27;9(6):e80641. doi: 10.1371/journal.pone.0080641 (PMC4074047; doi:10.1371/journal.pone.0080641)
Supplement: Table S2 — Estimates of parameters from three-population IMa2 analysis. (DOC) [file pone.0080641.s004.doc]

Table S2. Estimates of parameters from three-population IMa2 analysis for nuclear data only and combined mitochondrial and nuclear data set. Note that *θ* (theta), *τ* (time since ancestral population splitting), and *m* (migration rate) are parameter estimates scaled by per gene mutation rate *μ* which is for nucleus equal to the geometric mean of the mutation rates of all loci. T, *C. taenia*; E, *C. elongatoides*; N, *C. tanaitica*. Combined mito-nuclear data include one mtDNA locus and all nine nuclear loci. The *m*-values on significant paths given by LRT are shown in bold. # indicates that posterior density does not reach low levels near either the upper or the lower limit of the prior; † indicates that HPD interval did not appear to be contiguous and the estimates are not reliable. MLE, Maximum Likelihood Estimates; HPD95Lo, the lower bound of the estimated 95% highest density interval; HPD95Hi - the upper bound of the estimated 95% highest density interval. Parameters *m*_from TN to E_ and *m*_from E to EN_ are not summarised.

| Dataset | *θ*_T_ | *θ*_E_ | *θ*_N_ | *θ*_TN_ | *θ*_TEN_ | *t*_TN_ | *t*_TEN_ | *m*_from T to E_ | *m*_from E to T_ | *m*_from T to N_ | *m*_from N to T_ | *m*_from E to N_ | *m*_from N to E_ |
| --- | --- | --- | --- | --- | --- | --- | --- | --- | --- | --- | --- | --- | --- |
| MLE (nuclear data) | 0.012 | 0.324 | 0.012 | 0.828 | 2.244 | 0.010 | 1.130 | 0.075 | 0.025 | 0.225 | **5.175** | 0.026 | 0.025 |
| HPD95Lo | 0.000 | 0.132 | 0.000 | 0.000 | 0.060# | 0.000# | 0.350 | 0.000 | 0.000 | 0.000 | 0.000# | 0.000 | 0.000 |
| HPD95Hi | 0.396 | 0.756 | 0.228 | 21.610 | 23.990#^†^ | 15.550 | # | 34.770 | 24.020 | 43.170 | 41.770# | 23.270 | 36.230 |
| MLE (mito-nuclear data) | 0.175 | 0.435 | 0.175 | 1.155 | 0.255 | 0.105 | 1.445 | 0.005 | 0.005 | 0.205 | 0.105 | 0.045 | 0.005 |
| HPD95Lo | 0.065 | 0.195 | 0.065 | 0.000^†^ | 0.045 | 0.025^†^ | 0.905^†^ | 0.000 | 0.000 | 0.000 | 0.000 | 0.000 | 0.000 |
| HPD95Hi | 0.385 | 0.795 | 0.375 | 9.345^†^ | 9.995^†^ | 6.415^†^ | 9.995^†^ | 0.445 | 0.285 | 4.025 | 3.855 | 0.505 | 0.795 |
