# Supplementary material for: Distinguishing between Incomplete Lineage Sorting and Genomic Introgressions: Complete Fixation of Allospecific Mitochondrial DNA in a Sexually Reproducing Fish (Cobitis; Teleostei), despite Clonal Reproduction of Hybrids
Source: PLoS One. 2014 Jun 27;9(6):e80641. doi: 10.1371/journal.pone.0080641 (PMC4074047; doi:10.1371/journal.pone.0080641)
Supplement: Table S3 — Protocols used for gene amplifications. (DOC) [file pone.0080641.s005.doc]

Table S3. Protocols used for gene amplifications. Note that total genomic DNA was extracted from fin-clips stored in 96% ethanol using a DNeasy Blood and Tissue Kit Qiagen GmbH, Hilden, Germany) according to the company’s protocols. PCR was performed in 25 μl reactions. PCR chemicals were provided by Top-Bio (Prague, Czech Republic).

| Double stranded PCR | *28S*, *Rag1* | *AtpB* | *Act-2, N2, N4, N6, Rhod, RpS7, Cytb* |
| --- | --- | --- | --- |
| Template DNA | 10 ng | 10 ng | 10 ng |
| Reaction Mix | 2.5 μl 10x PCR Blue buffer | 12.5 μl PPP Master Mix | 12.5 μl PPP Master Mix with 5 mM MgCl_2_ |
|  | 0.5 μM 5’ primer | 0.5 μM 5’ primer | 0.5 μM 5’ primer |
|  | 0.5 μM 3’ primer | 0.5 μM 3’ primer | 0.5 μM 3’ primer |
|  | 1 mM (1.5 mM for *28S*) Mg^2+^ | 2 mM MgCl_2_ |  |
|  | 0.4 mM PCR dNTP mix |  |  |
|  | 2.5 U Taq DNA p. Unis |  |  |
|  | 1.5 μl PCR enhancer^TB^ |  |  |
