# Supplementary material for: Distinguishing between Incomplete Lineage Sorting and Genomic Introgressions: Complete Fixation of Allospecific Mitochondrial DNA in a Sexually Reproducing Fish (Cobitis; Teleostei), despite Clonal Reproduction of Hybrids
Source: PLoS One. 2014 Jun 27;9(6):e80641. doi: 10.1371/journal.pone.0080641 (PMC4074047; doi:10.1371/journal.pone.0080641)
Supplement: Table S4 — Primers used in this study. (DOC) [file pone.0080641.s006.doc]

Table S4. Primers used in this study.

| Primer (5’→3’) | |  | |  | |  |
| --- | --- | --- | --- | --- | --- | --- |
| *Act-2* | Act2F: GCATAACCCTCGTAGATGGGCAC | | *28S* | | C120p: TTA TGA CTG AAC GCC TCT AAG | |
|  | Act2R: ATCTGGCACCACACCTTCTACAA | |  | | D12pr: TGA CTT TCA ATA GAT CGC AG | |
|  | (Touria et al. 2003) | |  | | (Chen et al. 2003) | |
| *AtpB* | ATPSbf1: CTGGAGGGVAAYGATTTHTACCATGAGATGAT | | *N2* | | KUL2AF: TTTGAAGAGCTGCAGGCTTTTAGC | |
|  | ATPSbr1: CGGGCACGGGCRCCDGGNGGTTCGTTCAT | |  | | KUL2AR: GTGTACCACTCACATGAGCATGCA | |
|  | (Jarman et al. 2002) | |  | | (own) | |
| *Rag1* | 1F: AGC TGT AGT CAG TAY CAC AAR ATG | | *N4* | | KUL4AF: GCGATGTTACATGTGCAATCC | |
|  | 9R: GTG TAG AGC CAG TGR TGY TT | |  | | KUL6AF: GATAGTGCCGGTGAAGGGGCATCG | |
|  | (Quenouille et al. 2004) | |  | | (own) | |
| *Rhod* | Rh545: GCA AGC CCA TCA GCA ACT TCC G | | *N6* | | KUL4AR: GATCGNTACACGGTGCGAGGGACC | |
|  | Rh1073r: CCR CAG CAC ARC GTG GTG ATC ATG | |  | | KUL6AR: ACAGCTCCATTCACATGATGGGT | |
|  | (Zaragüeta-Bagilsa et al. 2002) | |  | | (own) | |
| *RpS7* | S7RPEX1F: TGG CCT CTT CCT TGG CCG TC | | *Cytb* | | L 15267: AATGACTTGAAGAACCACCGT | |
|  | S7RPEX2R: AACTCGTCTGGCTT TTCGCC | |  | | ThrR: ACCTCCGATCTTCGGATTACAAGACCG | |
|  | (Chow and Hazama 1998) | |  | | (Briolay et al. 1998; Doadrio et al 2002) | |

LITERATURE CITED

Briolay J., N. Galtier, R. M. Brito, and Y. Bouvet. 1998. Molecular phylogeny of Cyprinidae inferred from cytochrome b DNA sequences. Mol. Phyl. Evol. 9:100–108.

Chen W.-J., C. Bonillo, and G. Lecointre. 2003. Repeatability of clades as a criterion of reliability: a case study for molecular phylogeny of *Acanthomorpha* (Teleostei) with larger number of taxa. Mol. Phyl. Evol. 26:262–288.

Chow S., and K. Hazama. 1998. Universal PCR primers for S7 ribosomal protein gene introns in fish. Mol. Ecol. 7:1247–1263.

Doadrio I., J. A. Carmona, and A. Machordom. 2002. Haplotype diversity and phylogenetic relationships among the Iberian barbels

(*Barbus*, Cyprinidae) reveal two evolutionary lineages. J. Hered. 93:140–147.

Jarman S. N., R. D. Ward, and N. G. Elliott. 2002. Oligonucleotide primers for PCR amplification of coelomate introns. Mar. Biotechnol. 4:347–355.

Quenouille B., E. Bermingham, and S. Planes. 2004. Molecular systematics of the damselfishes (Teleostei: Pomacentridae): Bayesian phylogenetic analyses of mitochondrial and nuclear DNA sequences. Mol. Phyl. Evol. 31:66–88.

Touriya A, M. Rami, G. Cattaneo-Berrebi, C. Ibanez, S. Augros, E. Boissin, A. Dakkak, and P. Berrebi. 2003. Primers for EPIC amplification of intron sequences for fish and other vertebrate population genetic studies. BioTechniques 35:676–682.

Zaragüeta-Bagilsa R., S. Lavouéb, A. Tillierc, C. Bonillob, and G. Lecointreb. 2002. Assessment of otocephalan and protacanthopterygian concepts in the light of multiple molecular phylogenies. C. R. Biologies 325:1191–1207.
