# Supplementary material for: Distinguishing between Incomplete Lineage Sorting and Genomic Introgressions: Complete Fixation of Allospecific Mitochondrial DNA in a Sexually Reproducing Fish (Cobitis; Teleostei), despite Clonal Reproduction of Hybrids
Source: PLoS One. 2014 Jun 27;9(6):e80641. doi: 10.1371/journal.pone.0080641 (PMC4074047; doi:10.1371/journal.pone.0080641)
Supplement: Table S5 — PCR profiles used for gene amplifications in this study. (DOC) [file pone.0080641.s007.doc]

Table S5. PCR profiles used for gene amplifications in this study. Note that amplified DNA was purified using QIAquick PCR Purification Kit (Qiagen), and the Actin (*Act-2*) PCR fragment was extracted from the gel with QIAQuick Gel Extraction Kit (Quiagen), and direct cycle-sequenced with ABI PRISM Big Dye Terminator Cycle Sequencing Kit 1.1 (Applied Biosystems, Foster City, California, USA).

| PCR profile |  |
| --- | --- |
| *AtpB* | *N2, N4, N6* |
| 2’ at 94°C | 3’ at 95°C |
| 35 × (20’’ at 94°C, 60’’ at 45°C, 60’’ at 72°C) | 35 × (1’ at 94°C, 1’ at 55°C, 1’ at 72°C) |
| 06’ at 72°C | 10’ at 72°C |
| *Rhod* | *RpS7, 28S* |
| 5’ at 94°C | 5’ at 95°C |
| 35 × (1’ at 94°C, 1’ at 50°C, 90’’ at 72°C) | 1 × (1’ at 94°C, 90’’at 60–56ºC (2ºC/2cycle), 2’ at 72°C) |
| 7’ at 72°C | 30 × (1’ at 95°C, 90’’ at 54°C, 2’ at 72°C) |
| *Rag 1* | 7’ at 72°C |
| 5’ at 95°C | *Cytb* |
| 1 × (1’ at 94°C, 90’’at 62–56ºC (2ºC/2cycle), 2’ at 72°C) | 1’ at 95°C |
| 30 × (1’ at 95°C, 90’’ at 54°C, 2’ at 72°C) | 34 × (30’’ at 94°C, 30’’ at 50°C, 60’’ at 72°C) |
| 7’ at 72°C | 10’ at 72°C |
| *Act-2* |  |
| 3’ at 94°C |  |
| 34 × (1’ at 94°C, 1’ at 58°C, 20’’ at 72°C) |  |
| 10’ at 72°C |  |
