# Supplementary material for: Distinguishing between Incomplete Lineage Sorting and Genomic Introgressions: Complete Fixation of Allospecific Mitochondrial DNA in a Sexually Reproducing Fish (Cobitis; Teleostei), despite Clonal Reproduction of Hybrids
Source: PLoS One. 2014 Jun 27;9(6):e80641. doi: 10.1371/journal.pone.0080641 (PMC4074047; doi:10.1371/journal.pone.0080641)
Supplement: Text S1 — Detailed description of results from IM and IMA2 analyses. (DOC) [file pone.0080641.s009.doc]

**Results**

two-population IM model

Figure 5A-H and Supplementary Table S1 summarize the detail results of all IM runs. The marginal posterior density curves of divergence time *t* started from zero and rose to clear peaks, but their upper tails did not approach zero over the wide prior range. The ancestral population sizes (*θ*_A_) had flat posterior probability distribution for species pairs that were in the reciprocal monophyly at mitochondrial or nuclear loci. Therefore, the 95% intervals for *t* and *θ*_A_ did not appear to be contiguous.

For the nuclear datasets, we found symmetrical gene flow between *C. taenia* and *C. tanaitica* that was significantly greater than zero according to LRT. The MLE for migration rates from *C. elongatoides* to either *C. taenia* or *C. tanaitica*, were zero, while MLE for migration from either into *C. elongatoides* were positive, but the highest posterior density intervals always included zero value and were not significantly better than zero-migration model according to LRT.

For mitochondrial data, we observed two peaks of the curve profiles for migration and for the split time (*t*) from *C. elongatoides* to *C. tanaitica* when analyzing either all *C. tanaitica* individuals, or the western *C. tanaitica* sub-dataset only. One peak was located at zero and second peak (MLE) was greater than zero (Figure S2). The program was apparently not able to distinguish between scenarios of recent split with no migration and older split with higher migration. In result, the likelihood-ratio test did not reject the zero migration. When analyzing *C. elongatoides* and the eastern *C. tanaitica* sub-dataset, we found significantly positive MLE value for the migration into the eastern subpopulation of *C. tanaitica*, whereas zero migration was not rejected in the opposite direction. The migration for other species pairs were at zero. The results suggest an influence of the geographic structure in *C. tanaitica*.

three-population IMa2 model

Figure 6A-F and Supplementary Table S2 summarize the detail results of all IMa2 runs. As above, the highest posterior density intervals for the parameters of the two ancestral population sizes and split times did not appear to be contiguous.

Single locus analyses of mtDNA were not consistent among IMa2 runs and therefore, the results were excluded from the study.

The combined data set of nuclear DNA and mtDNA showed that the mean MLE value for the migration rates between *C. taenia* and *C. elongatoides* was at zero. The migration rates between *C. taenia* and *C. tanaitica* was greater than zero. The MLE for migration between *C. elongatoides* and *C. tanaitica* was asymmetrical with a zero value into *C. elongatoides*, but greater than zero into *C. tanaitica*. However, all highest posterior density intervals of the migration rates included zero and likelihood ratio test did not reject the zero-migration rate model for all of the migration parameter estimates. Nonetheless, we investigated the distribution of the number of migration events in the sampled gene genealogies separately for each locus and found 2 migration events related to the mtDNA locus from *C. elongatoides* to *C. tanaitica* and 23 migration events at nuclear loci, of which 20 events were from *C. taenia* and *C. tanaitica* and 3 in the opposite direction.

Altogether, the IM and IMa2 results suggested significant gene flow between *C. taenia* and *C. tanaitica* in nuclear DNA, although a direction of migration events remains ambiguous. The mitochondrion indicated a contrasting pattern, in which gene flow might have occurred from *C. elongatoides* to *C. tanaitica*.
